# The Missing Piece: The Structure of the Ti_3_C_2_T_*x*_ MXene and Its Behavior as Negative Electrode in Sodium Ion Batteries

**DOI:** 10.1021/acs.nanolett.1c02809

**Published:** 2021-09-23

**Authors:** Chiara Ferrara, Antonio Gentile, Stefano Marchionna, Irene Quinzeni, Martina Fracchia, Paolo Ghigna, Simone Pollastri, Clemens Ritter, Giovanni Maria Vanacore, Riccardo Ruffo

**Affiliations:** † Dipartimento di Scienza dei Materiali, Università degli Studi di Milano Bicocca, via Cozzi 55, 20125 Milano, Italy; ‡ National Reference Center for Electrochemical Energy Storage (GISEL)- Consorzio Interuniversitario Nazionale per la Scienza e Tecnologia dei Materiali (INSTM), via Giusti 9, 50121 Firenze, Italy; § Ricerca sul Sistema Energetico - RSE S.p.A., Via R. Rubattino 54, 20134 Milano, Italy; ∥ Dipartimento di Chimica, Università degli Studi di Pavia, via Taramelli 12, 27100, Pavia, Italy; ⊥ INSTM, Consorzio Interuniversitario per la Scienza e Tecnologia dei Materiali, via Giusti 9, I-50121 Firenze, Italy; # Elettra-Sincrotrone Trieste, 34149, Basovizza, Trieste, Italy; ∇ Institut Laue-Langevin, 71 avenue des Martyrs CS 20156, 38042 Grenoble, Cedex 9, France

**Keywords:** MXene, Ti_3_C_2_T_*x*_, structure, diffraction, extended defects, sodium ion batteries, XAS, Faults

## Abstract

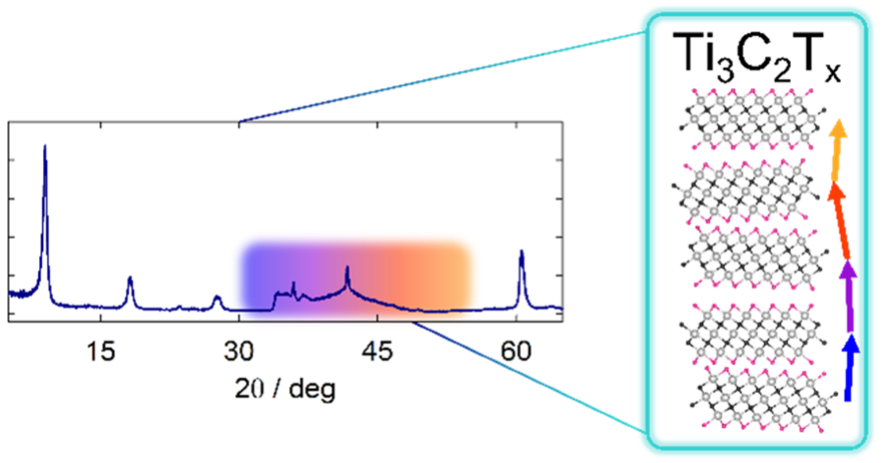

The most common MXene composition Ti_3_C_2_T_*x*_ (T = F, O) shows outstanding stability as anode for sodium ion batteries (100% of capacity retention after 530 cycles with charge efficiency >99.7%). However, the reversibility of the intercalation/deintercalation process is strongly affected by the synthesis parameters determining, in turn, significant differences in the material structure. This study proposes a new approach to identify the crystal features influencing the performances, using a structural model built with a multitechnique approach that allows exploring the short-range order of the lamella. The model is then used to determine the long-range order by inserting defective elements into the structure. With this strategy it is possible to fit the MXene diffraction patterns, obtain the structural parameters including the stoichiometric composition of the terminations (neutron data), and quantify the structural disorder which can be used to discriminate the phases with the best electrochemical properties.

The interest in the MXene family of 2D materials is booming thanks to the unique structural and functional properties.^1,2^ MXene compounds are described by the general formula M_*n*–1_X_*n*_T_*x*_ where M is a d-block transition metal (Sc, Ti, Zr, Hf, V, Nb, Ta, Cr, Mo...), X can be C, N, B, and T are termination groups (−O, −OH, −F, −Cl, −Br) which are usually undetermined in composition and stoichiometry.^1,3^ MXenes are generally obtained by the corresponding MAX precursor through the etching of the A element (Al, Sn). However, despite this chemical flexibility, about 70% of the reported experimental data concern the Ti_3_C_2_T_*x*_ composition since it can be obtained in various forms through accessible preparation routes.^1,4,5^ The peculiar functional properties make the MXenes new promising materials for their exploitation in several fields such as catalysis,^6^ gas storage,^7^ sensing,^8^ drug delivery, and cancer therapy,^9^ adsorption of heavy metals and radioactive pollutants,^10^ and electrochemical energy storage.^1,4,5,11^ The Ti_3_C_2_T_*x*_ is also the most appealing compound as anode material for Li–,^12^ Na–,^13^ and K–^14^ rechargeable batteries and supercapacitors.^15^ Beside the huge number of studies regarding the explorations of new compositions, substitutions, doping, hierarchic structures, and composites,^3,16^ some fundamental aspects of the structure of Ti_3_C_2_T_*x*_ MXene are still unaddressed. Indeed, (i) the composition and stoichiometry of the terminations are not completely controllable by the synthesis or postsynthetic treatments; (ii) the actual composition is difficult to determine; and (iii) the overall structure of Ti_3_C_2_T_*x*_ has not been described yet. A full clarification of these structural aspects is the missing step in the understanding of the correlation between functional properties and the different synthetic routes and treatments.^5,17−22^ Moreover, the understanding of the specific features can be the starting point for the improvement of the properties by controlling stoichiometry and structure.

One of the most interesting applications of the Ti_3_C_2_T_*x*_ phase is as a negative electrode in sodium ion batteries (NIBs), especially considering that state-of-the-art hard carbon materials show poor cyclability and low rate capability.^23^ The reversible intercalation of Na^+^ ions in nondelaminated Ti_3_C_2_T_*x*_ flakes was first demonstrated in 2015^24^ and then optimized by our group.^13^ We here report the long-term cyclability performance of two Ti_3_C_2_T_*x*_ MXenes obtained by different etching conditions from the Ti_3_AlC_2_ parental compound (MAX in the following): the Ti_3_C_2_T_*x*_ obtained in 30% HF for 5 h (MXT-30) and the Ti_3_C_2_T_*x*_ obtained in 5% HF for 24 h (MXT-5).

The two samples show some significant differences in sodiation/desodiation profiles (Figure 1a): MXT-5 presents a high voltage small plateau (2.3 V) not observed in MXT-30, which has been ascribed to the peculiar structural features of the structure.^24,25^ Moreover, the first cycle efficiency and the specific capacity are notably different, resulting in different degrees of irreversibility.^24^ To evaluate the practical applications in NIBs and to better highlight the difference in the electrochemical performances, we have tested the two materials in half cells for 530 cycles (Figure 1c). The MXT-5 and MXT-30 show average efficiencies at low current of 99.74% and 99.56%, respectively, while the capacity retentions are 100% and 95%. To our knowledge, the reversibility of the MXT-5 is the highest reported so far for Na-ion negative electrodes. A full cell has also been assembled using Na_0.44_MnO_2_ as positive electrode. The cell exploits the full MXene capacity, and it does not suffer of capacity fading after 150 cycles (Figure 1b). The MXT-5 not only has better cycling properties but also shows a higher rate capability, as already shown by our group, being able to deliver 85 mAhg^–1^ at 1500 mAg^–1^.^13^ It is clear that the electrochemical performance largely depends on the preparation routes, in turn directly affecting composition of terminations, and the degree of crystallinity, that is, the structure and morphology of the samples. It is thus essential to get insight in the short- and long-range structure of the MXene compounds to explain these relevant differences in the functional behavior.

**Figure 1:**
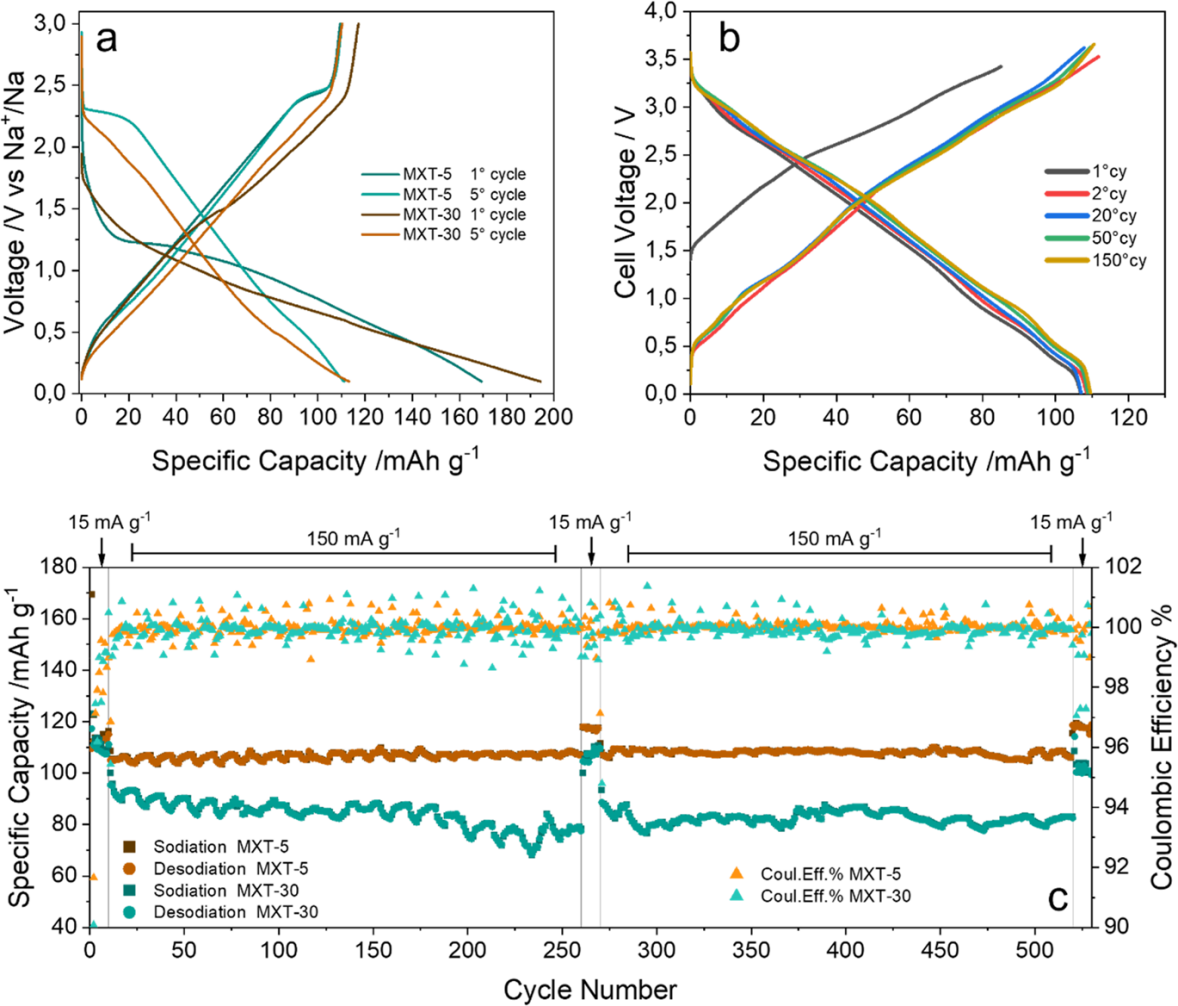
Electrochemical performances of Ti_3_C_2_T_*x*_ MXenes in NIBs. (a) MXT-5 and MXT-30 charge–discharge profiles at 15 mAg^–1^ in half cell versus metallic Na using 1 M NaPF_6_ EC-DEC as an electrolyte; (b) cell potential profile of a MXT-5 based NIB (galvanic chain MXT-5|1 M NaPF_6_ EC-DEC |Na_0.44_MnO_2_) at 15 mAg^–1^ (both specific capacity and gravimetric current are calculated on the MXT-5 mass); (c) long-term cycling of MXT-5 and MXT-30 in half cell versus metallic Na. The applied cycling protocols are 10 cycles at 15 mAg^–1^, 250 cycles at 150 mAg^–1^, 10 cycles at 15 mAg^–1^, 250 cycles at 150 mAg^–1^, and 10 cycles at 15 mAg^–1^.

Hereafter, we propose for the first time a model to describe the MXene structure and composition. A multitechniques approach has been implemented combining diffraction (neutron, X-ray), X-ray absorption (XAS), and transmission electron microscopy (TEM) experiments with previous results of X-ray photoelectron spectroscopy (XPS) and density functional theory (DFT) calculations obtained on the same materials by our group.^13^ XAS, XPS, and DFT results were combined to obtain the local short-range order (Ti–C and Ti–T connectivity), the terminations composition (−O, −F), and the most stable T-sites, which were used to build up a MXene structural model. This was the starting point for the analysis of the diffraction data to address the long-range order of Ti_3_C_2_T_*x*_ for which, up to now, no structural model (e.g., no space group, no unit cell) has been proposed. The validation of this approach was performed on the MAX phase precursor whose structure is well-known and already reported,^1^ while the analysis of TEM images was used to confirm the results. The comprehensive analysis and the fitting of the diffraction patterns is based on the recently proposed approach incorporated in the Faults software, a proper tool to treat layered and nonlayered materials characterized by the presence of staking faults.^26−30^ The model describes the structure without using a space group but instead introducing layers as the fundamental repeating units with associated stacking vectors and vector probability (Figure 2 and Section 4, Supporting Information (SI)).

**Figure 2:**
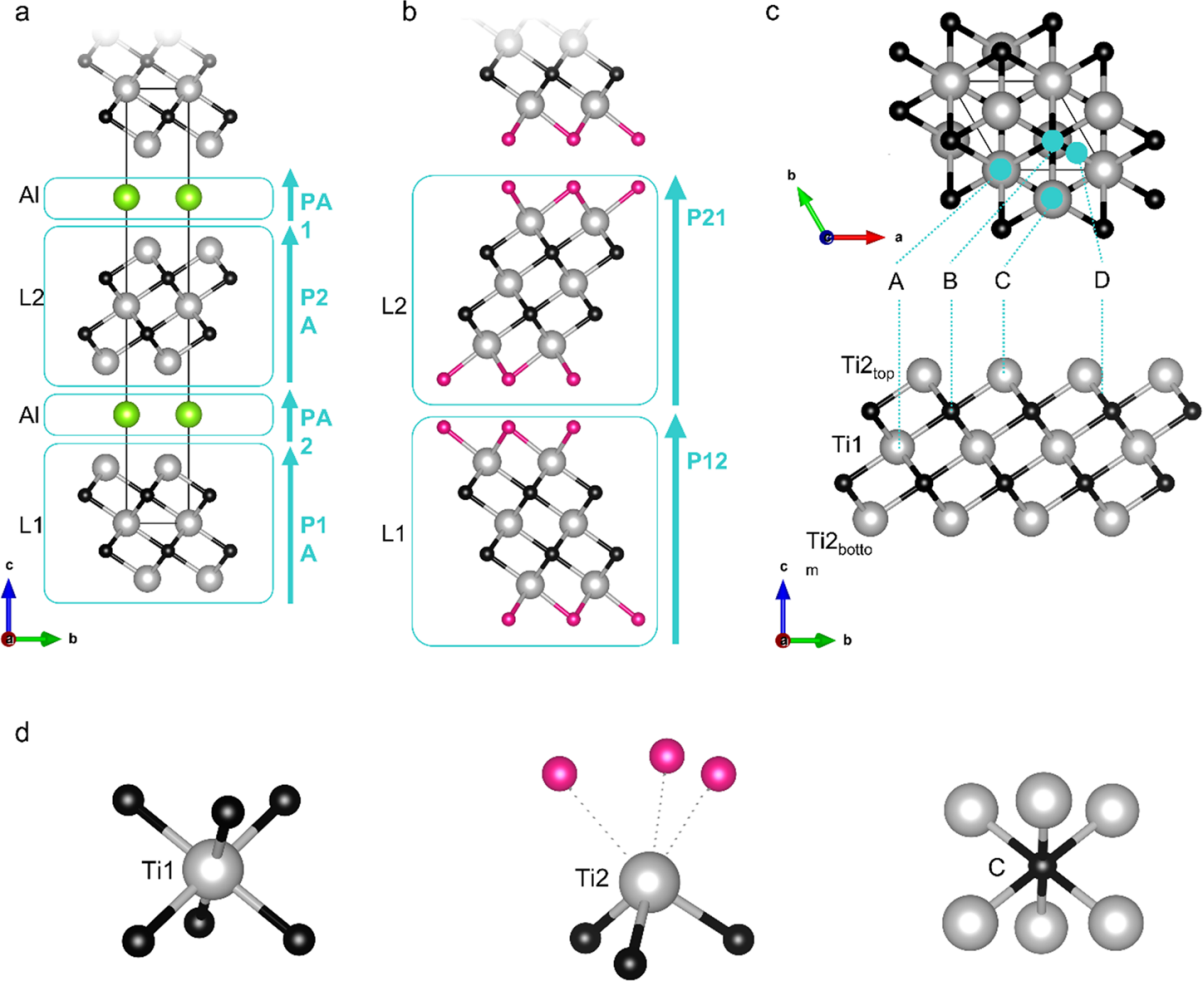
Picture of Ti_3_AlC_2_ and Ti_3_C_2_T_*x*_ (Ti gray, C black, Al green, T pink). (a) Ti_3_AlC_2_ MAX phase layered structure (SG *P*6_3_/*mmc*). In Faults description, the layered structure (...L1–Al– L2–Al...) is defined by the stacking probability vectors P1A, PA2, P2A, PA1 (light blue arrows). (b) Ti_3_C_2_T_2_ MXene layered structure derived from the MAX phase. In Faults description, the stacking sequence of layers (...L1–L2–L1...) is defined by the stacking probability vectors P12 and P21 (light blue arrows). (c) A,B,C,D termination sites derived from previous studies:^34−38^ Site A (coordinates 0, 0, *z*) is aligned with the vertical projection over the Ti1 site; site B (2/3, 1/3, *z*) is on the top of the carbon site; site C (1/3, 2/3, *z*) is on the top of the Ti2 site; site D (defined also as “bridging”) is halfway between the Ti2 and carbon sites. (d) Schematic representation of the first neighbor shell for Ti1, Ti2, and carbon sites. For Ti1 and carbon sites the positions of the nearest neighbors are defined; for the Ti2 site the location of the terminations (marked with dotted lines) is unclear. In the present scheme, the terminations are reported on the less energetic site (A) according to literature.^34−36^ For the specific phases reported in this work, optimization of the synthesis conditions has been carried out to ensure that all the Al is removed, and the synthesis can be considered completed. Indeed, both XPS and EDX analysis confirm the absence of Al residues in both the MXT-5 and MXT30.^13^

Despite the large abundancy of diffraction data of MXenes, their quantitative analysis is an open challenge, and the crystal structure of the MXene is still unresolved. Highly crystalline samples have been obtained only with specific techniques (e.g., epitaxial thin film^31−33^), but powdered materials are poorly crystalline.^2,20^ Patterns reported in literature are always characterized by features associated with the MAX precursor (space group *P*6_3_/*mmc*) as some reflections are characteristic of both of the systems.^1,13,21,22^ The atomic arrangements of Ti_3_AlC_2_ and Ti_3_C_2_T_*x*_ (Figure 2a,b) are strongly related to that of the MAX phase due to the peculiar synthesis^1,34−38^ (see Sections 1 and 2, SI for details on synthesis and structural features).

The analogies and the differences between the two systems can be also inferred from the relative diffraction patterns (Figure 3a,b and Figure S1). For the MAX phase a complete indexing of the pattern is possible; on the contrary for the MXenes, only the 00*l* and the 110 reflections are identified in analogy with the MAX phase. The downshift of the 002 reflection is used to monitor the variation of the interlayer distance due to the replacement of the A element with the terminations and the possible intercalation of ions/molecules. While the other 00*l* reflections are often clearly visible and resolved, the reflections in the 30–50° angular range (X-ray, Cu–K_α_ radiation) are blurred and merged in a single unresolved diffraction band (Figure 4a,b) which is always observed for the Ti_3_C_2_T_*x*_ compositions and other MXenes.^4,13,21,39^ The main structural differences between Ti_3_C_2_T_*x*_ and its MAX precursor are related to the replacement of the A site with the termination groups, whose chemical nature and sites are unknown and synthesis dependent. This info is also necessary to complete the MXene layers definition (L1–L2) in the Fault approach (Figure 2). For this reason, we first investigated the MXT-5, MXT-30, and MAX local electronic and atomic structure through XAS at the Ti K-edge. The XANES (X-ray absorption near edge structure) spectrum of the MAX phase displays a close resemblance to that of TiC and a close edge-energy position (Figure 3c). Conversely, the spectrum of both the MXene compositions is shifted to higher energies of about 1.5 eV with respect to Ti_3_AlC_2_ (Figure 3c), indicating a lower electron density on Ti due to the formation of bonds with electronegative atoms (F, O), in agreement with our previous results,^13^ and confirming the actual composition as Ti_3_C_2_F_2–*x*_O_*x*_. The composition is determined by the HF concentration: lower or higher concentrations lead to dominant O or F terminal groups, respectively. As a matter of fact, the aqueous etching does not allow for obtaining pure compositions (Ti_3_C_2_O_2_ or Ti_3_C_2_F_2_), and the accurate definition of the local and average F/O ratio is difficult to determine, as already reported in literature.^1,31−33^ The information from the different techniques exploited by our group (XPS, DFT, XAS) are considered as the starting point for the refinement of the relative F/O occupancies in the subsequent analysis of the neutron data. The extended X-ray absorption fine structure (EXAFS) analysis was used to determine the local structure around Ti1 and Ti2 atoms, allowing for the identification of the most populated termination sites. Although the creation of a cluster for the T1 is straightforward, the definition of the Ti2 coordination sphere requires to test the different possible termination sites (Figure 2d). After considering three structural models (see Section 4, SI), each composed by a TiC skeleton with different termination sites (Figure 2c), the results of EXAFS fitting (Figure 3d,e) suggest the that the A site is the most populated, followed by the B site, while the C site is less favorable (Tables S2 and S3), in agreement with DFT studies.^34−37^

**Figure 3:**
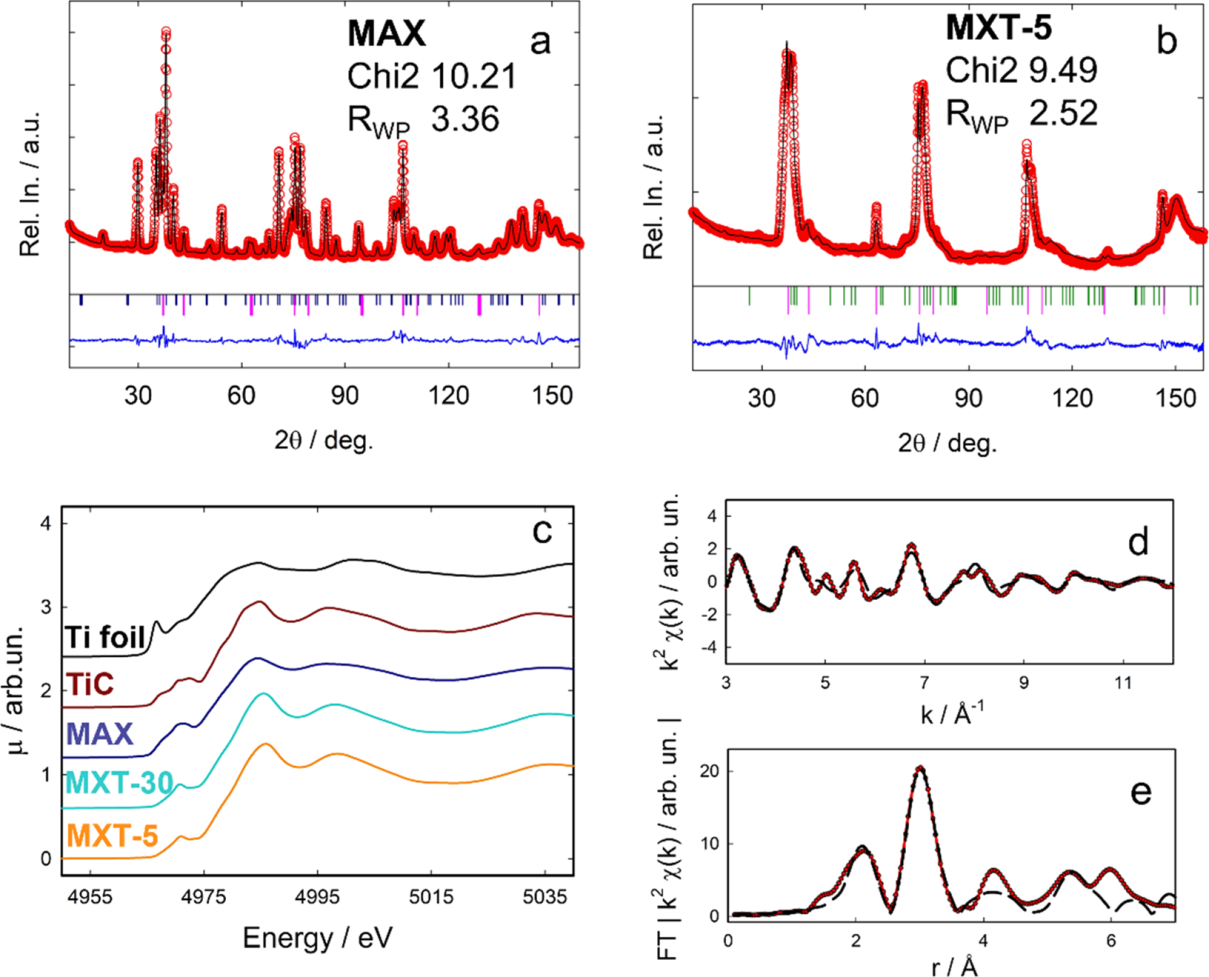
Neutron diffraction patterns for the (a) MAX and (b) MXT samples together with the results of Faults minimization and agreement factors (experimental data: red dots, calculated patterns black lines, difference functions: blue lines). Vertical bars are related to impurities: TiC pink, Ti_2_AlC blue, TiO_2_ green. (c) XANES spectra at the Ti K-edge for metallic Ti foil, TiC, MAX phase, MXT-5 and MXT-30. All the spectra are shifted along the *y*-axis for better clarity. The direct comparison of the spectra of MAX and MXT-5 without any vertical shift can be found in the SI, Figure S2. (d) EXAFS signal and (e) corresponding Fourier Transform (FT) of MXT-5 (experimental data, red lines; simulated curves (Model A), black dotted lines).

**Figure 4:**
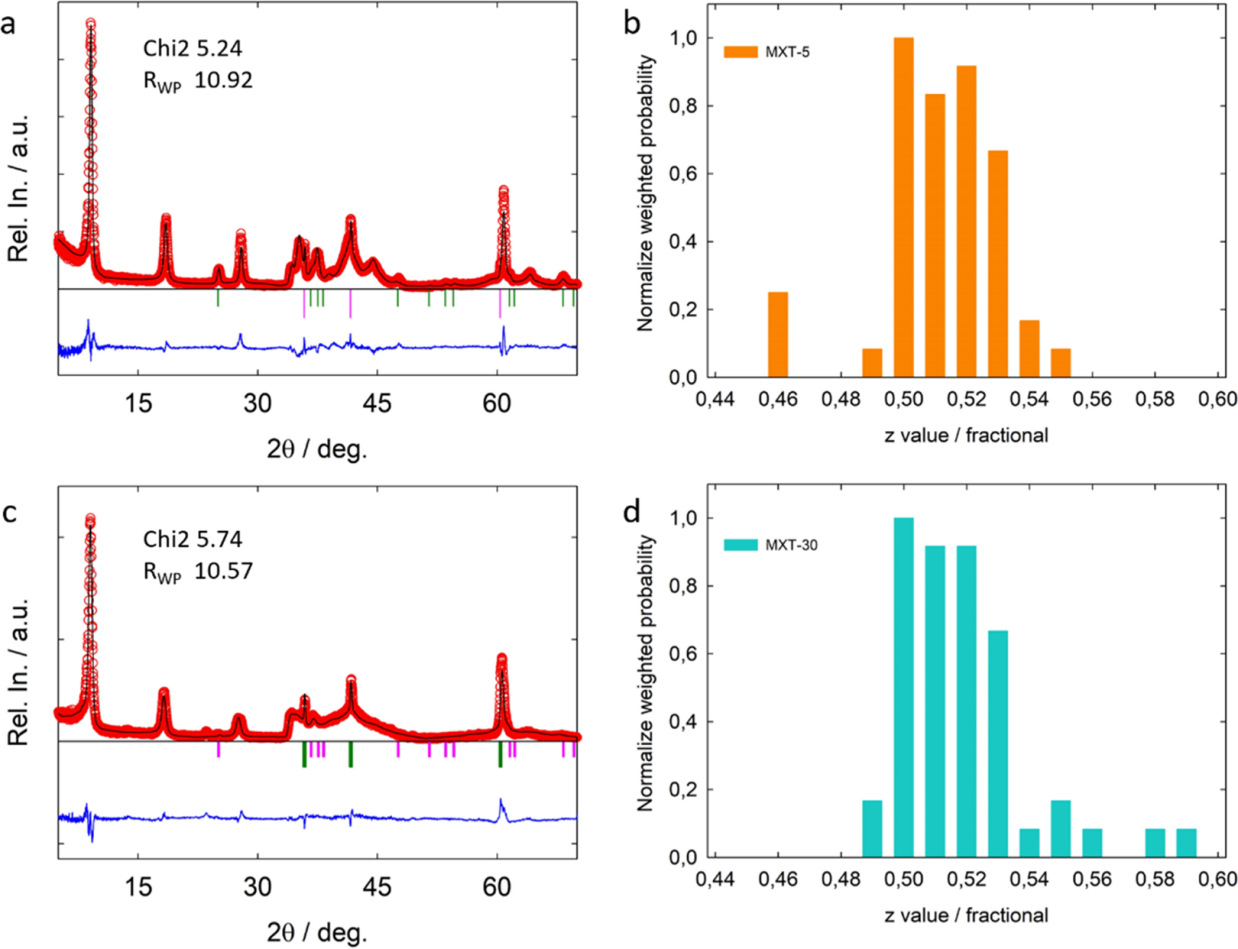
XRD data and Faults fit for the (a) MXT-5, and (c) MXT-30 samples. Normalized weighted population of *z*-components of the stacking vectors P12/P21 for the (b) MXT-5 and (d) MXT-30 obtained from the analysis of the Faults fits.

The use of Faults to describe the Ti_3_C_2_F_2–*x*_O_*x*_ structure was first validated analyzing the diffraction patterns of the Ti_3_AlC_2_ MAX phase, and the obtained results were compared with those derived from conventional Rietveld refinement (Section 3, SI, Figure S1, and Table S1). The MAX structure can be described as a sequence of [−L1–Al–L2–Al−] staking layers; L1 and L2 units show the same Ti–C connectivity but different orientation (see Figure 2a). The composition, structure, and relative orientation of each layer can be easily derived from the traditional space group-based description. The results of Faults minimization are reported in Figure 3a, and Figure S1 for neutron and X-ray diffraction data; based on the evaluation of the agreement factors, it is evident that the traditional Rietveld refinement and the Faults minimization give equivalent description of the structure and phase composition (see Table S1 and Figure S1). In particular, neutron powder diffraction data have been exploited as more sensitive to light atoms and thus allowing for the refinement of atomic position and relative occupancies of terminations and carbon sites. The presence of stacking faults and intergrowth in the MAX phase has also been considered trough Faults refinements (Section 3, SI); however, the results exclude the presence of significant levels of extended defects in good agreement with the description of the MAX as a layered compound with the maximum possible degree of ordering (MPDO).^40^ Thus, the only possible stacking sequence has been identified as [L1–L2]; this was used as a starting point for the description of the Ti_3_C_2_F_2–*x*_O_*x*_ long-range order in MXT-5 and MXT-30. Indeed, the complete removal of the Al layers has been demonstrated by our previous XPS and EDX analysis for both the specific compositions here discussed;^13^ these results are transferable as the complete removal of the A element of the MAX phase that can be obtained after careful optimization of the synthesis conditions.

The information obtained with the different techniques discussed so far has been considered to build the structural model (details in Section 4-SI) for the analysis of the diffraction data for MXT-5 and MXT-30. As already pointed out, indeed, the broadening of the diffraction patterns and the lack of a structural model (space group) does not allow the profile matching or Rietveld refinement for both neutron and X-ray diffraction patterns and the analysis here reported has been performed only with the Faults approach. The two building blocks of the MXene structures are the L1 and L2 units (Figure 2b) with associated stacking vectors P12 and P21. Terminal sites have been added in the L1 and L2 blocks, exploring different possibilities. The starting value for the *d*-interlayer spacing was defined by the evaluation of the interplanar distance associated with the position of the 002 reflection and subsequently refined. The *d*-spacing is strictly correlated with the *z*-component of the P12 and P21 stacking vectors that can be thus used as descriptors of the disorder in the structure. The effect of the variation of all the structural parameters (i.e., the positions of atoms M, X, T, composition, site occupancy, stacking vectors, and stacking probability) was evaluated by means of Faults simulation and minimization. To account for the *hk* band in the 30°–50° range, two major possible sources of disorder have been identified: the distribution of termination sites and the stacking disorder. The relative occurrence of A, B, and C termination sites with mixed F/O occupancies was allowed to vary, and the results indicated that the A sites, followed by the B site, are the most represented, while the C occupancy spontaneously reaches negligible values in excellent agreement with the results from XAS and DFT analysis.^13,34,35^ This was considered as the best starting model for the introduction of stacking faults, whose presence has been considered introducing extra L1 and L2 layers for which the stacking vectors were moved from the ideal position (0, 0, 0.5). Thanks to the implementation of these models and the Fault approach, it was possible to fit the experimental patterns of neutrons (Figure 3b) and X-rays (Figure 4a,c), thus obtaining for the first time experimental data relating to the structural parameters, the phase composition, the occupation of the termination sites, and their chemical nature (Table S3). In particular, the MXT-5 neutron pattern allowed the determination of the F/O ratio and to obtain the Ti_3_C_2_F_1.4_O_0.6_ composition.

The qualitative analysis of the results indicate that high levels of disorder are present already in MXT-5 obtained under milder condition. A quantitative analysis of the refined stacking vectors for the most represented sequences reveals that the stacking faults are affected for the *xy* and *z*-dimensions (see Figure 5a). In particular, the most represented values for the in-plane sliding are (1/3; 2/3), (2/3; 1/3) and their possible combinations. Considering that the A termination site is the most populated, this implies that the subsequent layers tend to glide for minimizing the repulsion between facing terminations and to assume an indented interlocked zipper-like configuration; this kind of dislocation is often associated with *z*-values of the P stacking vectors <0.5 and a reduction of the interlayer distance. The MXT-5 sample is characterized by minimum and maximum values of the refined *z*-parameter corresponding to 8.7 and 10.3 Å, respectively giving a weighted mean value equal to 9.5(0.4) Å (Figure 4b). The situation is even more severe for the MXT-30, presenting a broader *hk* band and an even wider distribution of *z*-values of the stacking vector with minimum and maximum values of 8.4 and 11.2 Å, respectively, and a weighted mean value of 9.8(0.5) Å (Figure 4d). From the diffraction point of view, the combined effect of the glide in the *xy* plane and of the distribution of interlayer distances hinders the coherent scattering among adjacent layers, resulting in the broadening of the (10*l*) class of reflection and thus generating the *hk* band, which is characteristic of the MXenes. Thus, while the distribution of the termination sites modulates the relative intensities of the *hk* features, the stacking faults determine the size of the broadening.

**Figure 5:**
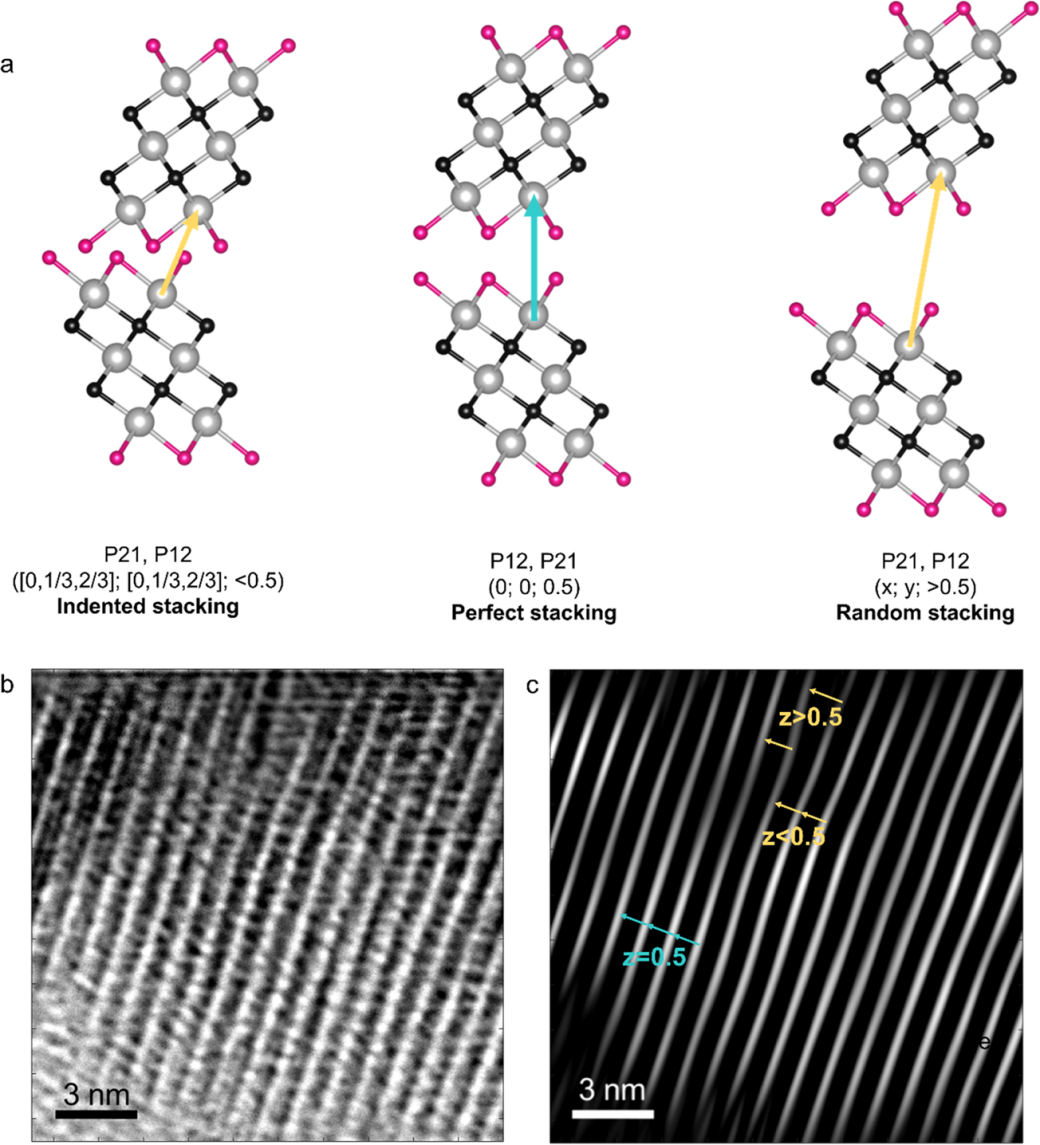
(a) Schematic representation of the most represented type of disorder depending on the observed value for the *z*-component of the stacking vector. The *z* = 0.5 represents the ideal crystal situation with the Ti1 of one layer directly facing the Ti1 of the adjacent layer (see arrow in the central panel). When *z* < 0.5, to minimize the repulsion among the termination, the sliding of adjacent layers of (1/3, 0) (2/3, 0) (0, 1/3), (0, 2/3) favor the indented configuration of facing Ti1 atoms. On the contrary, when *z* > 0.5 the loosening of the weak interactions leads to random sliding in the *x*, *y* direction of the adjacent layers. This type of defect can be detected in the TEM images obtained for the MXT-5. (b) TEM image of MXT-5; the picture shows a 17 × 17 nm area of the sample perpendicular to the *c*-axis. (c) Fourier-filtered TEM image of MXT-5, the picture shows the same area of the TEM image.

This scenario is also confirmed by TEM images (Figure 5b and Figure S6). To highlight the microscopic structure of the flakes along the stacking direction, we have implemented a Fourier filtering procedure (Section 1, SI) where only the sharp peaks due to the regular (00*l*) structure were selected to reconstruct a real space image (Figure 5c). The average *d*_002_ distance is 9.8 Å, which is in excellent agreement with the results obtained from XRD and neutron diffraction data analysis and with previous literature TEM reports.^41−43^ A closer inspection of the images shows a variability of such distance not only among different layers but also for the same neighboring layers along different spatial regions, especially at the edges of the MXene flakes, a situation which is, again, more severe from a qualitative point of view for MXT-30 (Figure S6). The measured interlayer distances and the obtained distributions (Figure S7) are in agreement with the results of the Faults minimizations (Figure 4b,d), highlighting the higher level of disorder for the MXT-30 compound. A wide range of previously reported HRTEM images for Ti_3_C_2_T_*x*_ samples obtained under similar synthetic conditions^41,44^ or as thin films and single layer^32,37^ show the same kind of extended defects inducing the *d*_002_ variability, which can be also affected by the presence of residual H_2_ or H_2_O molecules from the synthesis as also evaluated theoretically.^35^

The combined use of different investigation techniques addressing both the short- and long-range structure of the considered samples has made possible to highlight the similarities and differences among the MXT-5 and MXT-30 MXenes. In particular, the Ti–C skeleton and the electronic structure are very similar, while the differences reside in the termination compositions and the degree of disorder. The *z*-component of the stacking vector, which can be obtained by the Fault minimization, can be used as the quantitative descriptor of the structural disorder, and to discriminate between MXene compositions obtained under different synthetic conditions.

The structural features, the composition and the peculiar morphology concur in modulating the electrochemical behavior toward sodium intercalation. The nature of the termination (−F or −O) influences more the average electrical potential of sodium intercalation than the specific capacity, being related to the T-ion interaction and thus the Faradaic or capacitive behavior, as demonstrated by XPS and DFT calculations.^13^ The broader distribution in the interlayer distance of the MXT-30 sample can be associated with the higher amount of trapped H_2_ and H_2_O molecules (as widely discussed in ref (13)) with respect to MXT-5. This, combined with the higher surface area due to the open-lamellar structure, can be identified as the source of the higher irreversibility in the first cycle and in the lower capacity retention observed for MXT-30 with respect to MXT-5 (Figure 1a). A similar correlation among the higher degree of crystallinity and better stability has already been pointed out for layered materials.^45^ Thus, the overall outstanding reversibility of the MXT-5 as Na ion intercalation material can be associated with the favorable distance among the layer and the presence of stacking faults, providing a mechanism for the relief of the stress upon sodiation and desodiation cycles, as already highlighted for similar layered compounds.^46^

## Supporting Information Available

Brief discussion on the MAX and MXT structure, synthesis, and previous literature results on the structural characterization; information on the experimental methods and measurements are described; details and results from the XAS analysis, Rietveld refinement and models, faults minimization and simulations, building blocks for the MXene structure, and additional TEM images (PDF)
