# Supplementary material for: The Missing Piece: The Structure of the Ti3C2Tx MXene and Its Behavior as Negative Electrode in Sodium Ion Batteries
Source: Nano Lett. 2021 Sep 23;21(19):8290–7. doi: 10.1021/acs.nanolett.1c02809 (PMC8517972; doi:10.1021/acs.nanolett.1c02809)
Supplement: Supplementary file 1 — nl1c02809_si_001.pdf [file nl1c02809_si_001.pdf]

The missing piece: the structure of the $Ti_3C_2T_x$ MXene and its behavior as negative electrode in sodium ion batteries.

Chiara Ferrara[a,b], Antonio Gentile[a], Stefano Marchionna[c], Irene Quinzeni[c], Martina Fracchia[d], Paolo Ghigna[d,e], Simone Pollastri[f], Clemens Ritter[g], Giovanni Maria Vanacore[a], Riccardo Ruffo[a,b]

a - Dipartimento di Scienza dei Materiali, Università di Milano Bicocca, via Cozzi 55, 20125 Milano, Italy

b - National Reference Center for Electrochemical Energy Storage (GISEL) - Consorzio Interuniversitario Nazionale per la Scienza e Tecnologia dei Materiali (INSTM), via Giusti 7, 50121 Firenze, Italy

c - Ricerca sul Sistema Energetico - RSE S.p.A., Via R. Rubattino 54 - 20134 Milano, Italy

d - Dipartimento di Chimica, Università degli studi di Pavia, via Taramelli 12, 27100, Pavia, Italy;

e - INSTM, Consorzio Interuniversitario per la Scienza e Tecnologia dei Materiali, via Giusti 9, I-50121 Firenze, Italy

f - Elettra-Sincrotrone Trieste, 34149, Basovizza, Trieste, Italy

g - Institut Laue-Langevin - 71 avenue des Martyrs CS 20156, 38042 Grenoble, Cedex 9, France;

1. Experimental

Synthesis

The *MAX phase* with Ti_3AlC_2 nominal composition has been produced via spark plasma sintering (SPS), a well-established method for the preparation of this class of materials[1,2]. Based on a solid-liquid reaction synthesis, SPS promotes complete densification for Ti_3AlC_2 at relatively low temperature and with shorter sintering time than conventional hot pressing; to sinter a 12g Ti_3AlC_2 massive disk each SPS treatment requires 15 min.

Ti/Al/TiC starting powders were mixed in 1:1:1.9 proportion (off-stoichiometric) to minimize the secondary TiC phase precipitation during the MAX phase formation[3]. After soft milling in a turbula shaker for 24 h, the powders have been heated at 1300 °C for 5 minutes with a heating rate of 80 °C/min under a mechanical load, with compressive load of 43 MPa, and with 300 mbar of Ar pressure in the SPS chamber.

$Ti_3C_2T_x$ MXene samples have been prepared starting from round discs of Ti_3AlC_2, which were grounded and sieved to obtain a particle size < 50 μm. 500 mg of the obtained powders have been stirred in 10 mL of HF (Sigma-Aldrich HF > 40 wt%, CAS 7664-39-3). A 5% solution in water for 24 hours in a Teflon beaker at room temperature was used for the preparation under mild condition (MXT-5), while 10 ml of HF 30% in water for 5 hours was used for the preparation under strong conditions (MXT-30). The reaction between the MAX phase and hydrofluoric acid is exothermic and generally produces a large amount of bubbles of H_2. The low acid concentration allows to avoid this effect and support the hypothesis of slower reaction kinetics with respect to higher acid concentration. The slow kinetics and slow evolution of gas should allow also to obtain a more ordered layered material with respect to the MXene obtained using high concentration of HF. The yield of the process, obtained by the weight ratio after and before the etching, was ~96%.

The synthesis of the same MAX phase and MXene compositions have been already reported under the same experimental conditions in our previous work[4]; the results reported there for SEM-EDX, thermal analysis, and XPS investigations are thus considered as reference data for the analysis of the samples presented here.

X ray diffraction - XRD data have been collected on a Rigaku Miniflex 600 in Bragg Brentano θ-2θ geometry with acquisition in the 5°-80° angular range, step size 0.02°, and with the use of Cu Kα radiation.

Neutron diffraction - Neutron powder diffraction data have been recorded at room temperature on the high-resolution powder diffractometer D2B situated at the Institut Laue Langevin, Grenoble, France, using a wavelength of λ=1.594 Å. Samples of 800 mg were measured inside vanadium cylinders of 6 mm diameter for 8 hours (MAX) and 5 hours (MXT-5)

Traditional Rietveld refinements have been carried out with the use of the FullProf software[5]; the following parameters have been allowed to vary during the refinements: zero, scale, background (by points), cell parameters, atomic positions, global isotropic displacement factors (XRD data) and isotropic displacement factors for each individual element (neutron data), profile parameters,

asymmetry profile parameter, anisotropic strain parameters (neutron data only). The presence of secondary phases has been accounted for explicitly during the refinement with a multiphase approach. For the refinements of both the data sets, the proper instrumental resolution function file was considered; this, in combination with the proper selection of the profile function (option npr = 7 in FullProf), allowed for the evaluation of microstructural effects[5].

The Faults software has been used to simulate and refine the diffraction data. Levenberg Marquardt (LMA) fit was considered to carry out the refinements[6,7]. The quality of the refinements has been evaluated by the R factor and χ^2. The presence of secondary phases has been accounted for as background component, with refinable scale factors parameters allowing for phase quantification; details for introduction of secondary phases are reported in the Faults manual[6]. The following parameters have been allowed to vary during the refinements: scale factor, zero, cell parameters, atomic positions, global (XRD data) and individual element (neutron data) isotropic displacement factors, profile parameters, stacking vectors components, stacking probabilities.

<u>TEM</u> - TEM investigations were performed at the Microscopy Platform of the University of Milano-Bicocca (Italy) using a JEOL JEM 2100P operating at 200 kV, equipped with a LaB_6 source, and exhibiting a nominal point resolution of 2.4 Å. The images were then recorded with a Gatan RIO CMOS camera. The synthesized samples were dispersed in a solvent and transferred via drop casting onto a Cu grid, covered by a thin (3-4 nm) amorphous Carbon membrane. Images were taken at room temperature at different magnifications, with the TEM operated in bright-field parallel imaging mode, and adopting an in-gap objective aperture. To remove diffuse scattering noise, high-resolution images were processed using the following method: i) Fast Fourier Transform (FFT) of the real-space image; ii) application of an adaptive noise-removal Wiener filter to the FFT pattern; iii) inverse Fourier transform of the filtered FFT to obtain the final real-space image.

<u>XAS</u> - XAS spectra were acquired at the Ti K-edge (4966 eV) at the XAFS beamline operating at the Elettra synchrotron radiation facility in Trieste, Italy. All the spectra were recorded at room temperature in transmission mode; for this purpose, a proper amount of sample (as to give a unitary edge jump in the absorption coefficient) was mixed with cellulose and pressed to pellet. Energy calibration was achieved by measuring simultaneously the absorption spectrum of a metallic Ti foil, placed in a second experimental chamber after the I1 ionization chamber. Standard spectra of Ti_2O_3 and TiC were also acquired. The ring current and energy were 200 mA and 2.4 GeV, respectively. A Si(111) double-crystal monochromator was used, ensuring high-order harmonic rejection by detuning the second crystal. A water-cooled Pt-coated silicon mirror was used to obtain vertical collimation of the beam. The X-ray signal extraction and analysis, including the linear combination fits, were performed by means of the Athena code, belonging to the set of interactive programs IFEFFIT[8,9]. For the X-ray absorption near structure (XANES) analysis, the raw spectra were first background subtracted using a straight line and normalized to unit absorption 800 eV above the edge, where the extended X-ray absorption fine structure (EXAFS) oscillations are no more visible. The EXAFS data analysis was performed employing the Excurve code[10] using a k^2 weighing scheme and full multiple scattering calculations. The goodness of fit (GOF) was evaluated by the F parameter:

$$F = 100 \cdot \sum_{i}^{N} \frac{(\chi_{i,exp} - \chi_{i,calc})^2}{\sigma_i}$$

Electrode preparation and Electrochemistry - Electrodes of MXT-5 and MXT-30 were obtained by mixing of the active material with super P carbon (Alfa Aesar CAS 1333-86-4) and poly(acrylic acid) (PAA) (Sigma-Aldrich CAS 9003-01-4) in the 80:10:10 ratio. The $Na_{0.44}MnO_2$ electrodes were obtained by mixing the active material with super P carbon and Polyvinylidene fluoride (PVDF) (Sigma-Aldrich CAS 24937-79-9) in the 80:10:10 ratio. Each electrode composition was mixed for 1 h at 1000 rpm with an IKA Ultra-Turrax T-50 Homogenizer using N-methyl-2-pyrrolidone as solvent. An aluminum foil (MTI, thickness 15 μm) was coated with 250 μm of slurry using a doctor blade. The obtained coating was dried for 12 h under vacuum at 120 °C, and then calendered. Disc electrodes were cut with a diameter of 10 mm. The mass load of active material was around 1.5-2 $mgcm^{-2}$ for MXenes and 4 $mgcm^{-2}$ for $Na_{0.44}MnO_2$.

Electrochemical stability tests for MXT-5 and MXT-30 were carried out in 2-electrode cells, *i.e.* CR2032 (Hohsen Corp.) coin cells, assembled in an argon atmosphere (H_2O <0.1 ppm, O_2 <0.1 ppm) in a glove box. Metallic sodium was used as a counter electrode and reference while the electrolyte, supported on a glass fiber separator (Whatman®), was 1 M $NaPF_6$ (Alfa Aesar CAS 21324-39-0) in ethylene carbonate (EC) (Sigma-Aldrich CAS 96 -49-1) / diethyl carbonate (DEC) (Sigma-Aldrich CAS 105-58-8) in 1:1 $\%_{vol}$ ratio.

The electrochemical tests were performed using a current of 15 mAg^{-1} in cycles: 1-10, 261-270 and 521-530 to compare the stability of the materials every 250 cycles at higher currents (150 mAg^{-1}). The instrument was a multichannel Bio-Logic VMP3. For a correct evaluation of the reversibility, the charge efficiency was calculated at 15 mAg^{-1}, as at lower currents the secondary reactions have more time to occur.

The full cell was cycled with a Hohsen HS 3E cell. The cell is assembled with a 3-electrode configuration, where $Na_{0.44}MnO_2$ is the positive electrode, MXT-5 the negative electrode and Na metal is used as reference electrode. To reduce the MXT-5 irreversibility in the first cycle, the electrode was stabilized by cycling it between 0.1 and 3 V *vs.* Na^+/Na for 10 cycles in a 2-electrode Swagelok® cell. The electrode was then recovered and cycled against $Na_{0.44}MnO_2$ in the Hohsen HS 3E cell. During the cycling, the potentials of both positive and negative electrodes were measured *vs.* the reference electrode together with the potential of the full cell ($E_{Na0.44MnO2}$ - E_{MXT-5}). The OCV for the MXT-5 *vs.* Na^+/Na was 1.7 V and for $Na_{0.44}MnO_2$ *vs.* Na^+/Na was 3.2 V. The mass ratio between MXT-5 and $Na_{0.44}MnO_2$ was approximately 1:2.2. This difference in mass is useful to balance the capacities of the two electrodes. The capacity ratio between MXT-5 and $Na_{0.44}MnO_2$ was approximately 1:1.1. The full cell was cycled using the GCPL2 routine of the EC-Lab software which allows the cut-off control of all interfaces. The settings were: $E_{Na0.44MnO2}$ *vs.* E_{MXT-5} between 0 and 5 V, MXT-5 *vs.* Na^+/Na between 0.1 V and 3 V, $Na_{0.44}MnO_2$ *vs.* Na^+/Na between 2 V and 3.8 V.

2. The MAX phase and MXene structures and synthesis

The MAX class of compounds includes more than 90 compositions[2,11], as the structure can accommodate different species, especially on the M and A sites, and the dimensionality of the M_{n-}

$_1X_n$ slabs can vary. Independently of the composition, the MAX phase is described by the $P6_3/mmc$ space group (n. 194), demonstrating that this system can accept high levels of substitutions[12-15]. The structure can be described as a compact Ti_3C_2 arrangement forming slabs running along the *ab* plane, and stacked perpendicularly to the *c* axis, alternating to the Al layers. Ti occupies two distinct crystallographic sites (Ti1 and Ti2), while only one site if found for C and for Al, respectively. Ti1 occupies the inner site within the layers and is coordinated to six C atoms, while the Ti2 species lay at the surface of the Ti_3C_2 slab. Ti2 atoms are coordinated to three C and three Al centers in a more distorted coordination with respect to the $Ti1C_6$. The C coordination sphere can, consequently, be described as $C(Ti1)_3(Ti2)_3$ while Al is found in an highly distorted environment even if it is coordinated formally only by the Ti2 species, $Al(Ti2)_6$ (see Figure 2). The MAX phases show a unique combination of properties (light weight, high strength and elastic modulus, high thermal and chemical stability, excellent thermal shock resistance, thermal and electric conductivity, low thermal expansion coefficient), deriving directly from the layered structure and specifically from the anisotropy of the bonding nature (M-X covalent bonds extending in the layers along the *ab* plane and M-A metallic bonds connecting different layers along the *c* direction)[1,16-18]. This feature, besides determining the exploitable functional properties, allows to produce $M_{n-1}X_n$ layered structures (MXenes) by selective removal of A slabs, exploiting the different reactivity of the M-X and M-A bonds. Generally, the MXene is obtained by acidic etching of the precursor MAX phase. In the specific case of the Ti_3AlC_2 composition, the etching step results in the $Ti_3C_2T_x$ phase, where T represents the possible terminations; the T species can be -F, -O-, =O, -OH, -Cl, that can be bound/coordinated with solvent and H_2O molecules or adsorbed atoms trapped within the layers[2,12].

The obtained structure is strictly related to that of the precursor, being based on 2D sheets with $M_{n+1}X_nT_x$ composition, and presenting an increased *c* lattice parameter[12,19,20]. The composition, distribution, and stoichiometry of the terminations can be, to some extent, controlled by the synthesis conditions (acid concentration[12,21], direct use of HF or exploiting in-situ HF generation[19,20], duration of the leaching treatment, use of molten salts[2] or the use of electrochemical etching[22]), sonication and temperature, and by post synthesis thermal treatments[4,12,14]. The various procedures reported in the literature differ in the duration of the treatment (generally above 4 h), acid concentration, nature of the acid species (fluorides are always needed but the source can be different; pristine HF or HF produced in situ have been exploited as well as bisfluorides salts such as $NaHF_2$, KHF_2, NH_4HF_2[23] aqueous solution or molten salt[11]), temperature of the treatment (above 35 °C)[24,25].

From the abovementioned description it is possible to infer some considerations regarding the MXene structure:

- As the synthesis of the MXene exploits the reactivity of the M-A bond under acidic condition while the M-X bonds are not affected, it must be assumed that the M-X connectivity is maintained moving from the MAX phase to the MXene.
- Due to the etching reactions (removal of Al with the use of HF and subsequent introduction of T terminations), the electronic structure of the compound is expected to significantly change; the oxidation states of Ti and C in MXene can be different with respect to those observed in the MAX phase. Thus, while the connectivity is the same, bond lengths can be different.

- M-T bonds are expected to be strong, replacing the M-A interactions. Contrary to the MAX phase where a M-A-M sequence is expected, in the MXene the M-T---T-M sequence is present.

3. MAX phase structural analysis – Rietveld refinements of X ray and neutron patterns, Faults models

The Rietveld refinements of the X-Ray and neutron diffraction patterns obtained for the MAX sample have been based on the structural model discussed in the previous section; results of the refinement are reported in Figure S1 and Table S1. To improve the initial model, the presence of TiC and Ti_2AlC has been considered and they have been quantified, amounting to ~7% and ~2%, respectively. Such impurities are frequently observed as secondary phases obtained during the synthesis of the MAX phases[4,26]. Overall, the structural description of the MAX compound is in good agreement with previous report on the same composition[27].

The peak shape was modeled with the TCH Pseudo Voigt profile function[28] both for X-Ray and neutron data refinement; this allows for the evaluation of the microstructure of the sample. The proper instrumental resolution file was considered for both the refinements, allowing for the separation of the instrumental and sample contribution to the peak broadening. The refinement of the strain related parameters significantly boosts the fit of the peak profile for both X-Ray and neutron data, while the refinement of size parameters just slightly improves the quality of the refinements. The isotropic strain evaluated from the neutron data refinement is 16.83(2)%%. The main effect of the strain parameters on the quality of the refinement, with respect to the size parameters, has been verified both for neutron and X-ray data. The strain value obtained from the X-Ray data is higher, but the data obtained from neutron diffraction are considered more reliable due to the higher quality and the constant scattering factor over the angular range. This residual strain can be related to the non-equilibrium conditions of the SPS synthesis. The independent refinements of the X-Ray and neutron data lead to similar results in terms of cell parameters, interatomic distances, and quantitative phase determination (Table S1), and is in good agreement with previously reported data on the same composition[27].

For Faults minimization, the layered structure of the MAX phase was modeled considering MX and A layers; the M, X, and A positions have been derived from the results of conventional Rietveld refinement. The stacking vectors associated to these layers are P1A, PA2, P2A, PA1 and are defined as (0, 0, 0.378), (0, 0, 0.5), (0, 0, 0.378), and (0, 0, 0.5) with associated probability 1, to account for the perfect structure. To confirm that this description is equivalent to those obtained using the $P6_3/mmc$ space group, both Rietveld refinement and Fault minimization have been carried out and the results were compared. To ensure a direct comparison between the two approaches, the background, displacement factors, and amount of secondary phases have been assumed as equal. Results of the minimizations are reported in Figure S1 and Table S1.

The presence of stacking faults can be explored moving the value of the stacking vector out of the equilibrium value (*i.e.* the perfect crystal structure): attempts to refine this parameter led to its original value. The presence of different stacking sequences (L1-Al-L1-Al, L2-Al-L2-Al, L1-L1, L1-L2) and

of layers of the lower and higher order MAX phases (Ti_2C and Ti_4C_3) have been considered, being the spark plasma sintering (SPS) synthesis compatible with the formation of such defects. The initial probability value of each defect was set equal to 10%; all the refinements lead to negligible probability values. Based on these results, the presence of significant levels of extended defects in the MAX sample can be excluded.

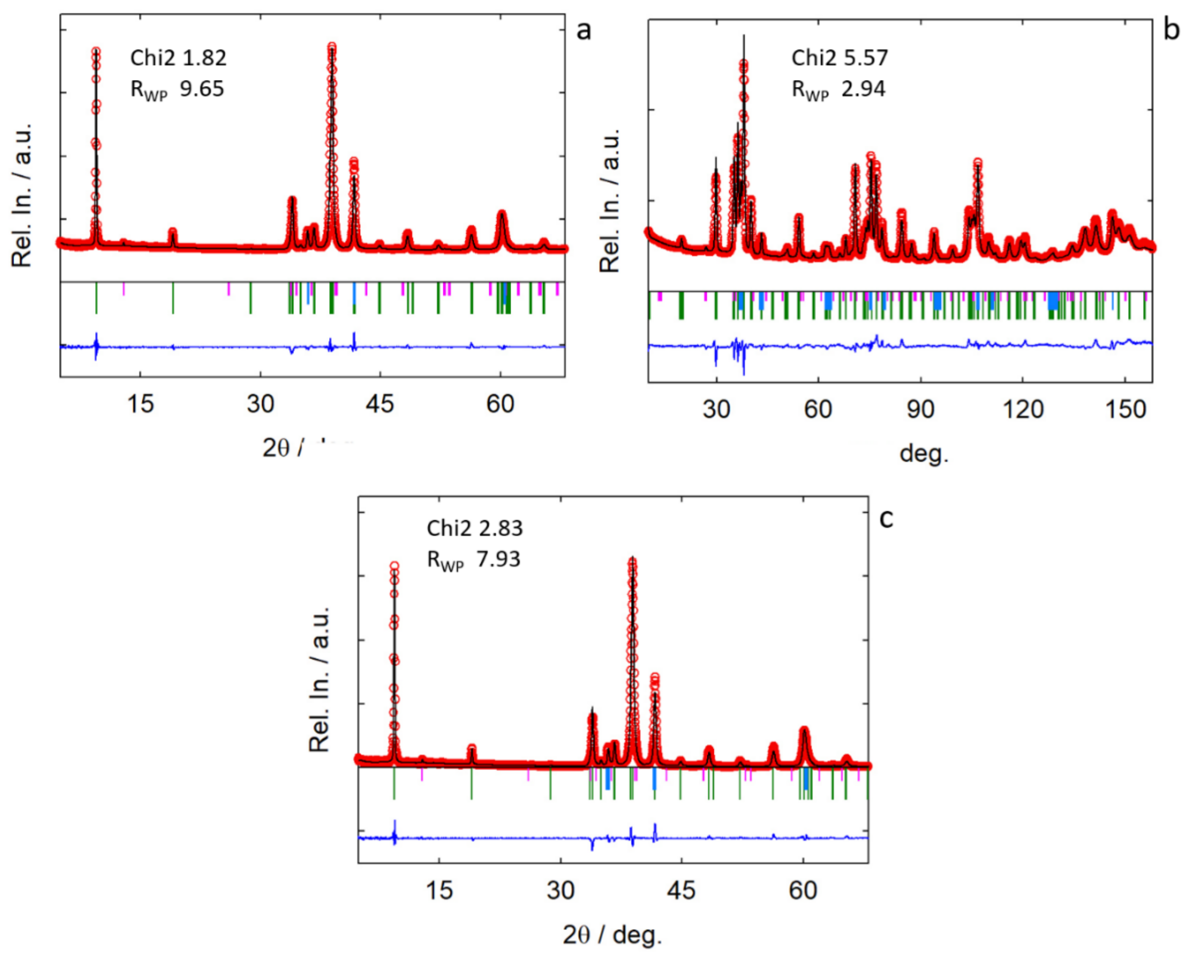

Figure S1 – Rietveld refinement for the XRD (a) and neutron diffraction (b), Faults analysis for XRD (c) patterns obtained for the MAX sample together with the obtained agreement factors.

	XRD		Neutron	
	Rietveld	Faults	Rietveld	Faults
**Cell parameters / Å**				
**a / Å**	3.0757 (3)	3.0742 (4)	3.0752 (4)	3.0759 (3)
**c / Å**	18.5888 (4)	18.5706 (8)	18.5804 (3)	18.5714 (5)
**Interatomic distances / Å**				
**Ti1-C / Å**	2.191 (3)	2.2051 (1)	2.1909 (5)	2.2151 (1)
**Ti2-C / Å**	2.080 (3)	2.0766 (2)	2.0771 (5)	2.0741 (1)
**Ti2-Al / Å**	2.8862 (9)	2.8767 (4)	2.8929 (4)	2.8612 (2)
**Phase composition / %**				
**Ti3AlC2**	90.66 (0.35)	91.162	90.85 (0.71)	89.750
**TiC**	7.31 (0.11)	6.053	6.87 (0.07)	8.232
**Ti2AlC**	2.03 (0.10)	2.785	2.27 (0.22)	2.018
**Agreement factors**				
**Chi2**	1.82	2.83	5.57	10.21
**Rwp**	9.65	7.93	2.94	3.36

Table S1 – Cell parameters, selected interatomic distances, phase composition, and agreement factor for the Rietveld and Faults analysis of X-Ray and neutron diffraction pattern analysis for the MAX sample.

4. MX phase structural analysis – Literature review and Faults models

As discussed in the previous sections, up to now no detailed description of the MXene structure has been reported. However, some points can be fixed from the review of the extensive literature pertinent to such compounds, and from considerations emerging from the analysis of the MXene synthesis. A brief discussion is here reported to clarify the basis on which the starting structural model for the MXene was derived, considering both literature data and the results from diffraction and XAS for the MAX sample.

TX layers - Due to the nature of the synthesis, exploiting the different nature of the T-A and T-X chemical bonds, the complete removal of A is expected, while the T-X connectivity is supposed to be unchanged moving from the MAX precursor to the MXene compound. Indeed, for the Ti_3AlC_2 composition, the removal of Al has been confirmed by different experimental techniques such as XRD[2,20] and XPS[29]. In particular, our previous work on the same samples confirmed this result[4]. For these reasons, in the structural model of the MXene no A layers have been introduced. At the same time the Ti-C network is maintained, defined by the pseudo hexagonal symmetry, in agreement with the XAS data discussed in the next paragraph. This has been confirmed by experimental studies based on pair distribution function (PDF) investigation[30,31], TEM images[32-35], and XAS[36].

The leaching process starting from the MAX phase is supposed to gradually remove Al from the A layers, and the Ti2 coordination sphere is restored with the introduction of T terminations, that re-establish the six-fold coordination. On these bases, changes in the stacking sequence are not expected

when passing from the MAX to MXT compounds. As no antiphase domains are present in the MAX sample, these defects are not expected in the MXT structure, that is thus described with a regular staking sequence of L1-L2-L1... On the contrary, stacking faults are expected due to the nature of the synthesis and the weakening of the interlayer interactions. The structure of the L1 and L2 layers is not supposed to be altered during the synthesis as the etching is selective for the Al species. Previous experimental (based mainly on TEM and SAED images)[12,23,37] and computational[38-40] studies indicate that both the composition and structure of the layers are maintained. Thus, it is still possible to define the layers with the use of the point group derived from the $P6_3/mmc$ space group. The L1 and L2 layers have been thus defined tacking the refined coordinates of the MAX sample from the refinements reported in Section 3.

Terminations: composition and sites - The removal of the A layers is compensated by the introduction of T terminations. Two major issues must be considered: the possible sites and the composition/stoichiometry of such terminal groups. As schematized in Figure 2c, different sites are possible; the evaluation of site location and stability has been assessed mainly with theoretical approaches, due to the experimental difficulties in evaluating these aspects. To get experimental insight, a combined analysis of XAS and diffraction data has been performed to validate the DFT calculations.

X-ray Absorption Spectroscopy (XAS)

X-ray absorptions near edge spectroscopy (XANES) data at the Ti K-edge are reported in Figure S2 for the MAX and MXT compositions allowing a direct comparison together with the Ti_2O_3 spectrum as reference.

The edge energy position was found to be coincident to Ti_2O_3 (Figure S2). However, due to the intrinsic differences in term of electronic structures between titanium oxides and carbides, a precise assessment of the formal oxidation state close to Ti(III) is only tentative. The pre-edge region is constituted by two peaks, clearly visible for the MAX phase, attributed to the Ti *1s* → C *2p* + Ti *3d* (t_{2g} and e_g) hybridized orbitals[41,42]. The peak at lower energies has a considerably lower intensity for MXene than for the MAX phase, as a consequence of their different electronic structure[43]. Only very slight differences are detected between MXT-5 and MXT-30 around the pre-edge peak (see Figure S2), confirming that their electronic and atomic structure is substantially the same.

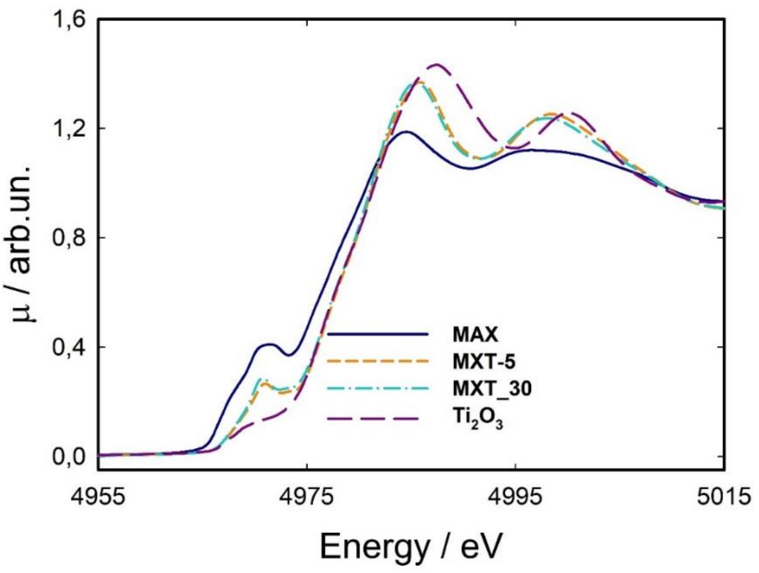

Figure S2: Normalized XANES spectra at the Ti K-edge of the samples MAX, MXT-5, MXT-30, and Ti_2O_3.

EXAFS fitting

MTX-5 was then taken as representative for further EXAFS (Extended X-ray absorption fine structure) analysis to investigate the local structure.

With respect to the F/O composition, the EXAFS analysis cannot be exploited to probe the relative occupancies to the very similar behaviour of the two elements while is sensitive to their termination position. For these reasons, three structural models with formula $Ti_3C_2F_2$ were considered, each composed by a TiC skeleton, but having three different termination positions derived from previous DFT calculations[44-46] (Figure 2c and S3).

The EXAFS signal was fitted starting from the $Ti_3C_2F_2$ structure having the *P*6₃/*mmc* space group. This structure is derived from the MAX phase, and it is composed by Ti_3C_2 slabs on the *ab* plane, and upper and lower Ti atoms which are directly bond to the F terminations. Ti occupies two distinct crystallographic sites (2*a* and 4*f* Wyckoff positions, respectively), so two clusters were used for the fit: one where Ti has occupancy of 0.33, accounting for Ti in the Ti-C plan, and one with occupancy 0.67, accounting for upper and lower Ti, bound to the terminations, and for the 1:2 multiplicity ratio of the Wyckoff positions. Three starting models were used for the fit: i) model A, with F in the 0, 0, *z* position (A site), model B, with F in the 2/3, 1/3, *z* position (B site), and model C with F in the 1/3, 2/3, *z* position (C site), where *z* was set to 0.2; this values was selected as representative of the Ti-O and Ti-F average distances. Representation of the layers with A, B, and C configurations are reported in Figure S3. Due to the consistent dimension of the starting model, the number of refined parameters was strictly controlled. The number of independent points (N_{idp}) can be obtained by the Nyquist criterion[47]:

$$N_{idp} \cong \frac{2\Delta k \Delta R}{\pi}$$

In our case, the number of refined parameters was 18, well below the N_{idp} value, which, with the Δk and ΔR ranges used in the fits, is equal to 34. Concerning the different contributions of the different atoms in the clusters to the EXAFS, we here note that the first peak in the EXAFS FT is due to the C atoms in the first coordination shell, while the second peak is due to the Ti atoms in the second coordination shell. The role of F atoms is more subtle, as they contribute to the peaks in the EXAF FT at distances larger than 4 Å via multiple scattering paths. These distant peaks contain several additional multiple scattering paths involving Ti and C atoms.

The best fit was obtained for model A, showing an F factor equal to 10%. The structural parameters obtained after the refinement are shown in Table S2. The fitting results for model B and C can be found in Figure S4. The interatomic distances obtained from the best fit (model A) are in excellent agreement with the same distances obtained from Faults minimization, performed on both X-Ray and neutron data. Concerning model B, the agreement between the experimental and the theoretical curves is worse than for model A (see Figure S4), but the overall fit is still acceptable with an F factor equal to 15%. Conversely, model C leads to a very bad agreement and a F factor equal to 20%. This is particularly evident in the EXAFS FT at r>4 Å, where the agreement for model C is considerably worse than for model A and B. These results point toward the fact that model A is very well representative of the MXene local structure, suggesting a preferential occupation of the A site with respect to the B site. As noted before, the distinctive geometry of the terminations in the C sites leads to a very bad agreement at long distances, and therefore the presence of F terminations in the C sites seems to be negligible, as theoretically predicted by DFT calculations[44-46].

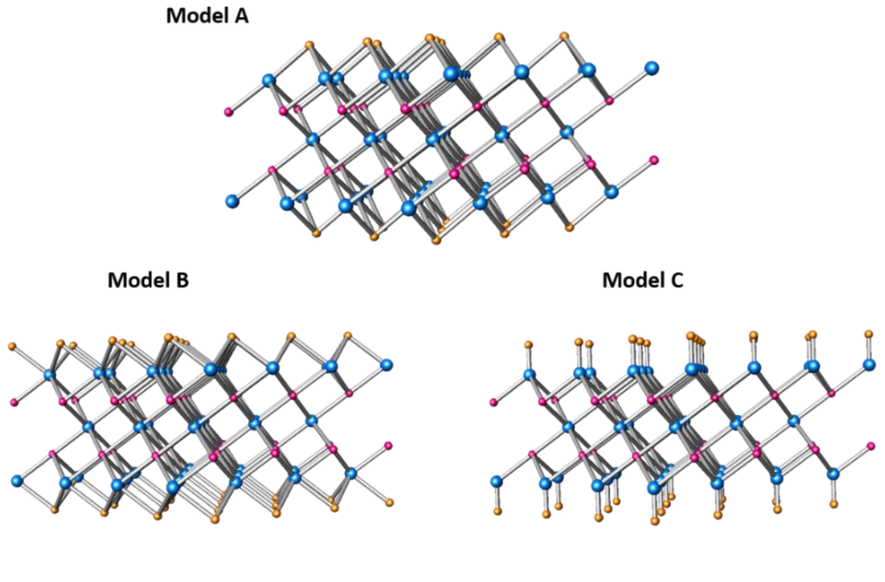

Figure S3: Representation of a single layer of the MXene $Ti_3C_2F_2$ structure for model A, B and C, differing for the position of the F terminations. Ti atoms are in blue, C in pink and F in orange.

Cluster 1 (occupancy 0.33)					
**Shell**	N	Atom	R(Å)	σ^2 (Å^2)	R (Å) crystallographic
**1**	6	C	2.13(1)	0.006(2)	2.168
**2**	6	Ti	3.02(1)	0.007(1)	3.061
**3**	6	Ti	2.98(4)	0.007(1)	3.072
**4**	6	C	3.751*	0.01(2)	3.751
**5**	2	F	3.926*	0.02(2)	3.926
**6**	6	Ti	4.336*	0.03(1)	4.336
**7**	12	C	4.841*	0.1(2)	4.841
**8**	12	F	4.978*	0.05*	4.978
**9**	6	Ti	5.301*	0.01(1)	5.301
**10**	12	Ti	5.308*	0.01(1)	5.308
**11**	2	F	5.889*	0.05*	5.889
**12**	6	Ti	6.121*	0(2)	6.121
**13**	12	C	6.494*	0.02(7)	6.494
**14**	6	F	6.597*	0.05*	6.597
**15**	6	F	6.597*	0.05*	6.597
**16**	12	F	6.638*	0.05*	6.638
**Cluster 2 (occupancy 0.67)**					
**Shell**	N	Atom	R(Å)	σ^2 (Å^2)	R (Å) crystallographic
**1**	3	C	2.13(1)	0.006(2)	2.168
**2**	3	F	2.50(4)	0.02(2)	2.263
**3**	6	Ti	3.02(1)	0.007(1)	3.061
**4**	3	Ti	2.98(4)	0.007(1)	3.072
**5**	3	C	3.751*	0.01(2)	3.751
**6**	1	C	3.769*	0.01(2)	3.769
**7**	3	F	3.806*	0.02(2)	3.806
**8**	3	F	3.811*	0.02(2)	3.811
**9**	3	Ti	4.336*	0.03(1)	4.336
**10**	1	Ti	4.790*	0.00(1)	4.790
**11**	6	C	4.841*	0.1(2)	4.841
**12**	6	C	4.855*	0.1(2)	4.855
**13**	6	F	4.884*	0.05*	4.884
**14**	3	F	4.888*	0.05*	4.888
**15**	3	Ti	5.301*	0.01(1)	5.301
**16**	1	Ti	5.301*	0.01(1)	5.301
**17**	6	Ti	5.307*	0.01(1)	5.307
**18**	3	Ti	5.327*	0.0(1)	5.327
**19**	6	Ti	5.684*	0.1(3)	5.684

20	6	F	5.767*	0.05*	5.767
21	6	Ti	6.121*	0(2)	6.121
22	3	Ti	6.143*	0(2)	6.143
23	6	C	6.494*	0.01*	6.494
24	3	C	6.504*	0.01*	6.504
25	6	F	6.526*	0.01*	6.526

Table S2: Structural parameters obtained after the EXAFS refinement with model A for MXT-5: N: coordination numbers (number of atoms in the shell) R: coordination distances; σ^2 : EXAFS Debye-Waller factors. The values of the distances marked with an asterisk are the crystallographic ones and were not included in the refinement. In model A, Ti occupies two distinct crystallographic sites, resulting in two different local chemical environments. Therefore, two clusters around Ti were used for the fit. Cluster 1 accounts for Ti in the Ti-C plan, with Ti in the 2a Wyckoff position of space group $P6_3/mmc$, while cluster 2 accounts for upper and lower Ti atoms bound to the terminations, with Ti in the 4f Wyckoff position. To account for the different multiplicities of the two sites, with the ratio 1:2, cluster 1 was weighted for 33%, while cluster 2 was weighted for 67%.

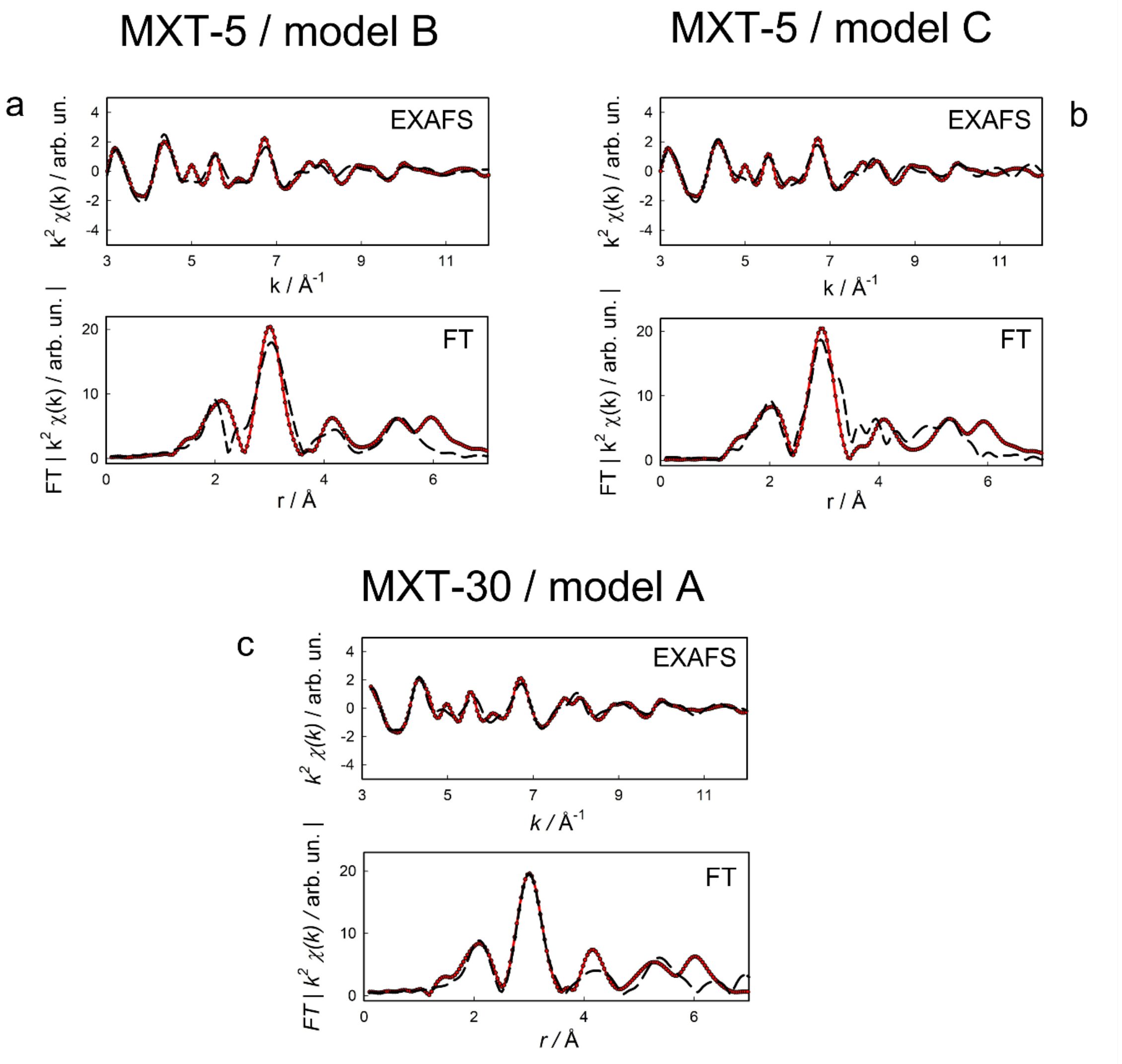

Figure S4: EXAFS signal and corresponding Fourier Transform (FT) of low MXene, fitted with model B (a) and model C (b) for the MXT-5; EXAFS signal and corresponding Fourier Transform (FT) fitted with model A for the MXT-30 (c). The red lines represent the experimental data, while the black dotted lines are the fits obtained with the two different structural models. Reported models are based on $Ti_3C_2F_2$ composition; analogue fitting results were obtained starting from the $Ti_3C_2O_2$ model structures instead of $Ti_3C_2F_2$.

Analogous results were obtained for MXT-30. The EXAFS fit obtained with model A is shown in Figure S4c, while Table S3 shows the refined structural parameters. It can be noted that the values of the distances and Debye-Waller factors are equal, within the experimental error, to those obtained for MXT-5.

**Cluster 1 (occupancy 0.33)**					
**Shell**	N	Atom	R(Å)	σ^2 (Å^2)	R (Å) crystallographic
**1**	6	C	2.15(2)	0.006(2)	2.168
**2**	6	Ti	3.03(1)	0.007(1)	3.061
**3**	6	Ti	3.02(1)	0.007(1)	3.072
**4**	6	C	3.751*	0.02(3)	3.751
**5**	2	F	3.926*	0.02(2)	3.926
**6**	6	Ti	4.336*	0.023(7)	4.336
**7**	12	C	4.841*	0.1(4)	4.841
**8**	12	F	4.978*	0.05*	4.978
**9**	6	Ti	5.301*	0.01(1)	5.301
**10**	12	Ti	5.308*	0.01(1)	5.308
**11**	2	F	5.889*	0.05*	5.889
**12**	6	Ti	6.121*	0(2)	6.121
**13**	12	C	6.494*	0.01(6)	6.494
**14**	6	F	6.597*	0.05*	6.597
**15**	6	F	6.597*	0.05*	6.597
**16**	12	F	6.638*	0.05*	6.638
**Cluster 2 (occupancy 0.67)**					
**Shell**	N	Atom	R(Å)	σ^2 (Å^2)	R (Å) crystallographic
**1**	3	C	2.15(2)	0.006(2)	2.168
**2**	3	F	2.46(9)	0.03(2)	2.263
**3**	6	Ti	3.03(1)	0.007(1)	3.061
**4**	3	Ti	3.02(1)	0.007(1)	3.072
**5**	3	C	3.751*	0.02(3)	3.751
**6**	1	C	3.769*	0.02(3)	3.769
**7**	3	F	3.806*	0.02(2)	3.806
**8**	3	F	3.811*	0.02(2)	3.811
**9**	3	Ti	4.336*	0.023(7)	4.336
**10**	1	Ti	4.790*	0.001(7)	4.790
**11**	6	C	4.841*	0.1(4)	4.841
**12**	6	C	4.855*	0.1(4)	4.855
**13**	6	F	4.884*	0.05*	4.884
**14**	3	F	4.888*	0.05*	4.888
**15**	3	Ti	5.301*	0.01(1)	5.301

16	1	Ti	5.301*	0.01(1)	5.301
17	6	Ti	5.307*	0.01(1)	5.307
18	3	Ti	5.327*	0.02(6)	5.327
19	6	Ti	5.684*	0.1(5)	5.684
20	6	F	5.767*	0.05*	5.767
21	6	Ti	6.121*	0(2)	6.121
22	3	Ti	6.143*	0(2)	6.143
23	6	C	6.494*	0.01*	6.494
24	3	C	6.504*	0.01*	6.504
25	6	F	6.526*	0.01*	6.526

Table S3: Structural parameters obtained after the EXAFS refinement with model A for MXT-30: N: coordination numbers (number of atoms in the shell) R: coordination distances; σ^2: EXAFS Debye-Waller factors. The values of the distances marked with an asterisk are the crystallographic ones and were not included in the refinement.

X-ray diffraction – simulated patterns

The problem of location of the terminations has been investigated computationally with two approaches: the identification of different sites (generally labeled according to the symmetry of the resulting packing, fcc and hcp or A and B[44]), and the identification of different configurations, *i.e.* the description of a layer with defined termination position on both $Ti2_{top}$ and $Ti2_{bottom}$ (generally called I and II[45,46]). Figure 2 summarizes the different possibilities reported in the literature. In the former case, the relative stability of the different single sites for the different termination species (O, F) is explored, while the latter considers the configuration of a single layer, with all the terminal groups (below and above the single layers) in a specific position. We accounted for all the possibilities, investigating the effect of different positions, different configurations and also mixed cases (presence of more than one terminal group on different possible positions and more than one possible configuration).

Previous DFT calculations converge in reporting that the relative energy scale of such sites, where the A position (fcc symmetry, on the top of T1 site) is the most favorable for the adsorption of terminations[44] and that the I configuration (all terminations on A sites) is the less energetic[45].

The termination-termination and surface-surface interactions are much weaker with respect to the termination-surface interaction, thus the dominant contribution is due to the effective termination site. Regarding the composition, the stability increases from OH to F to O. Terminal O results to be the most favorable species, and therefore dehydrogenation of the OH groups is expected upon aging. At the same time, it must be pointed out that in previous theoretical works, a simplified vision is proposed as the evaluation of the termination stability is performed for a single site[44,48], or for layers where all the terminations are represented on the same site[45]. Nevertheless, these extreme conditions of high ordering (a single site for the termination on top and bottom of the layers) as well as the end-members for the composition ($Ti_3C_2F_2$, $Ti_3C_2O_2$) are not expected experimentally. A distribution both in sites and composition is expected.

Regarding the occupation of the termination sites, functionalized MXenes are thermodynamically stable (negative free energy for the functionalization reaction)[49,50]. Thermodynamic calculations demonstrated that completely functionalized surfaces are more stable with respect to partially

terminated slabs. This can be rationalized considering the stabilization of metal species with full coordination: the MXene layers expose the Ti centers as outer species, and the six-fold coordination stabilize these centers[49,51]. Among different terminations, previous results demonstrate that the MXenes are prone to oxidation with the replacement of -F terminal groups with -O based species. Based on these considerations, is it possible to suppose that the occupancy of the termination sites is close to 1.

Calculations also reveal that a full coverage (stoichiometry $T_3X_2T_2$) is expected as the full oxidation is favored; thus, no bare Ti2 sites are expected[44,49]. Regarding the composition, no end members are obtained experimentally, but some trend can be outlined. The introduction of high levels of F are associated with the synthesis with high concentration of HF, while O and OH terminations are introduced by etching under milder conditions (low HF concentration, in situ HF generation)[4,20]. Finally, clustering of the terminations is not expected.

Thus, the terminations are expected to introduce a first source of disorder as different sites are present, and the possibility of mixed composition must be taken into account. For the modeling of the layers, all sites A, B, and C have been considered, as well as the presence of F and O chemical species. Different F/O occupancies do not influence the XRD pattern but can have an impact on neutron data. Thus, the relative F/O occupancies has been determined by the analysis of the neutron data and the obtained model has been used with no modifications for the analysis of the XRD data. As no evidence of ordering (*i.e.* extra reflection due to super-structures) are present, random distribution of F and O on the different sites has been considered.

Simulations of XRD patterns for highly ordered configurations based on all terminations on A, B, and C sites have been performed considering the perfect stacking sequence L1-L2 with P12 = P21 = (0, 0, 0.5); the results are reported in Figure S5. Fully ordered configurations have been explored for the composition $Ti_3C_2F_2$, using the same termination sites on the top and bottom of the layer (AA, BB, CC). As expected, the presence of terminations on well-defined sites leads to well-defined diffraction patterns; the different configurations are characterized by different patterns, but none of them can fit the experimental data. Super ordered systems (*e.g.* AB, BC, ABC etc. or regular alternation of A and B positions within the *ab* plane) have not been considered, as the presence of a super-lattice will lead to the appearance of extra reflections that are not observed experimentally.

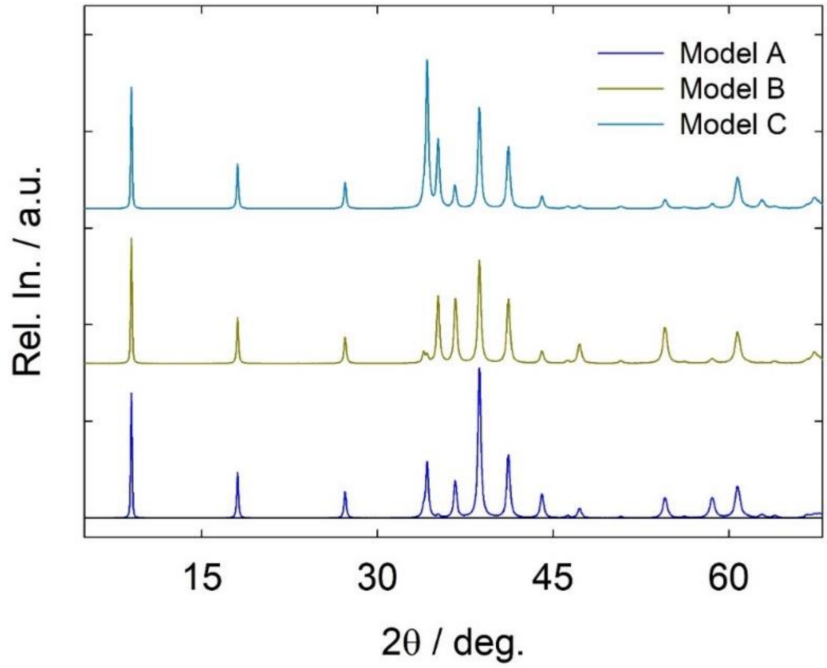

Figure S5 – Simulation of XRD pattern for the ideal, perfect crystal $Ti_3C_2F_2$ composition with no stacking disorder and all the terminations on the A site (blue pattern), on the B site (olive green pattern), on the C site (light blue pattern).

Stacking – The presence of different termination sites with mixed occupancies on the layer surface and weak interaction among the adjacent layers makes the presence of stacking faults highly probable. The presence of 2D defects can be thus invoked to explain the peculiar diffraction patterns of MXenes reported for all the compositions obtained by top-down approach[2,4,20]. The differences in the reported patterns, generally in the position of the 002 peak and in the degree of coalescence in the 30°-50° degrees range for the XRD data, can be related to the degree of stacking disorder. If the stacking disorder is low and/or the random displacements are small compared to the perfect stacking, the scattering coherence among the layers is not completely destroyed (in this case faults can be described as a fluctuation of the translation vectors about a mean value represented by the perfect crystal structure). Higher level of defects or completely random stacking faults have the effect of destroying the coherence among the different layers and, as a consequence, the resolution of the *hkl* reflections associated to these layers is weakened. When the presence of random stacking is systematic (turbostratic stacking) the coherence is completely lost and unresolved *hkl* reflections with strong anisotropy are observed, degenerating in *hk* diffraction bands[7,52,53]. Globally, the presence of a high level of 2D defects reduces or lowers the periodicity along specific directions; all the aspects of the diffraction pattern (peak position, shape, and broadening) can be affected by the nature, distribution and abundance of such defects. This hinders the use of conventional analysis techniques (*e.g.* Rietveld refinement) based on the assumption of the periodicity of the structure and thus of the scattering factors.

The description of non-periodic crystal containing stacking faults needs defining the nature of the stacking faults, *i.e.* translation vector, associated probability, and distribution. This approach is considered in the Faults software, which has already used for the description of layered systems[7,54-56], and used here as a key tool for the first attempt of the analysis of MXene diffraction patterns.

In summary, the main hypothesis for the building up of the MXene layered model are:

- No Al, no intergrowth, no mismatched layers (*e.g.* L1-L1, L2-L2) in MAX phase, as evident from previous XPS analysis and Faults minimization on the MAX composition. For the description of the MXene structure, two distinct layers with the same composition but different orientation are needed, L1 and L2, as reported in Figure 2. The M-X layers are built from the structural data obtained from traditional Rietveld refinement of XRD and neutron data, and confirmed by the Faults minimization and XAS data analysis: the pertinent structural data are reported in Table S1 and Table S2;
- Presence of A and B sites for termination, mixed occupancies for F and O on both sites based on previous XPS, DFT analysis on the same composition[4]. The relative occupancies of the sites (A and B) and composition (F and O species) have been allowed to vary;
- To account for the degree of disorder detected in the experimental patterns, a model composed of ten distinct layers was needed. The layers are all of the L1 and L2 type; differences are only in stacking vectors and associated probability (L1 = L3 = L5 = L7 = L9; L2 = L4 = L6 = L8 = L10); stacking vectors and probability were allowed to vary during the minimization. Attempts to obtain the same results in terms of agreement factors have been made based on model consisting of 2, 4, 6, and 8 layers, but no satisfying results have been obtained. The starting values for all the stacking vectors was the same, representing the perfect ideal situation (0; 0; 0.5). The stacking vectors and probabilities were refined in subsequent minimizations following the sequence (*z* value; *x* and *y* values, stacking probability);

	**XRD**	**Neutron**
**MXT-5**	Faults	Faults
**Cell parameters / Å**		
**a / Å**	3.0461 (2)	3.0443 (3)
**c / Å**	18.9875 (3)	19.0199 (4)
**Interatomic distances / Å**		
**Ti1-C / Å**	2.1357 (6)	2.1407 (3)
**Ti2-C / Å**	2.1342 (6)	2.1089 (3)
**Relative occupancy**		
**A/B sites**	0.8570 : 0.1430	0.7988 : 0.2012
**F/O ratio**	0.6834 : 0.3166*	0.6834 : 0.3166
**Phase composition / %**		
**$Ti_3C_2(F/O)_2$**	91.2	90.5
**TiC**	7.1	9.0
**TiO_2**	1.6	0.4
**Agreement factors**		
**Chi2**	5.24	9.49
**Rwp**	10.92	2.52

	XRD
**MXT-30**	Faults
**a / Å**	3.0498 (2)
**c / Å**	19.0688 (4)
**Ti1-C / Å**	2.1442 (7)
**Ti2-C / Å**	2.1447 (3)
**Relative occupancy**	
**A/B sites**	0.9640 : 0.0360
**F/O ratio**	0.5 : 0.5 *
	**Phase composition / %**
**$Ti_3C_2(F/O)_2$**	94.9
**TiC**	4.8
**TiO_2**	0.3
	**Agreement factors**
**Chi2**	5.74
**Rwp**	10.57

Table S4 – Cell parameters, selected interatomic distances, phase composition, and agreement factor for the Faults analysis of X-Ray and neutron pattern analysis for the MXT-5 and MXT-30 samples. (indicates that the F/O ratio has not been refined for the X-Ray data). For the MXT-5 the occupancy ratio was taken from the neutron refinement, for the MXT-30 was fixed 0.5:0.5).*

5. MXT-30 TEM images

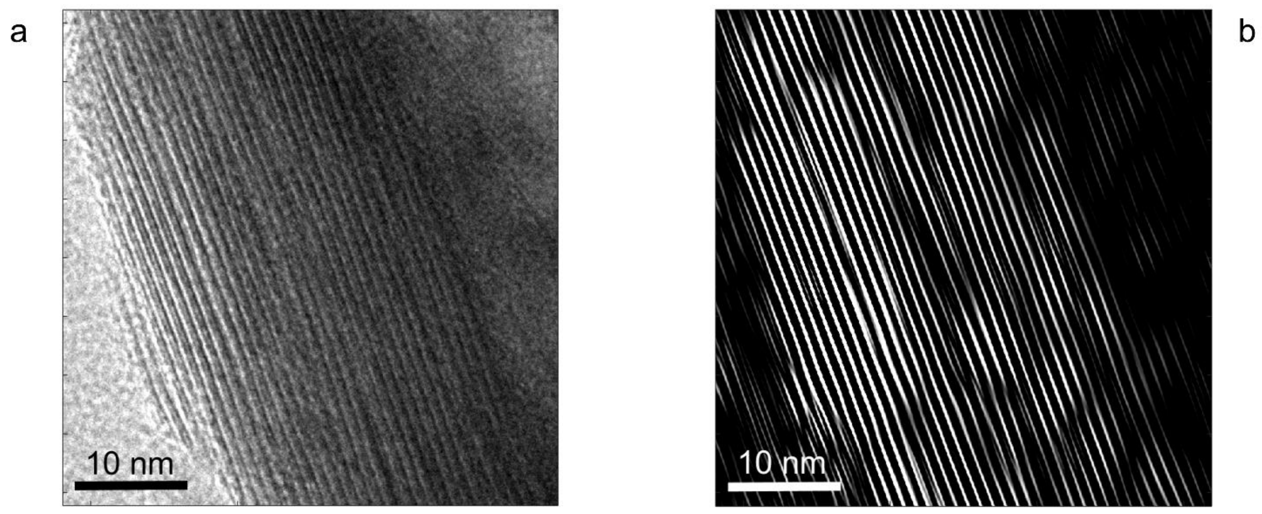

Figure S6 – (b) TEM image of MXT-30, the picture shows a 44x44 nm area of the sample perpendicular to the c axis; (c) Fourier filtered TEM image of MXT-5, the picture shows the same area of the TEM image.

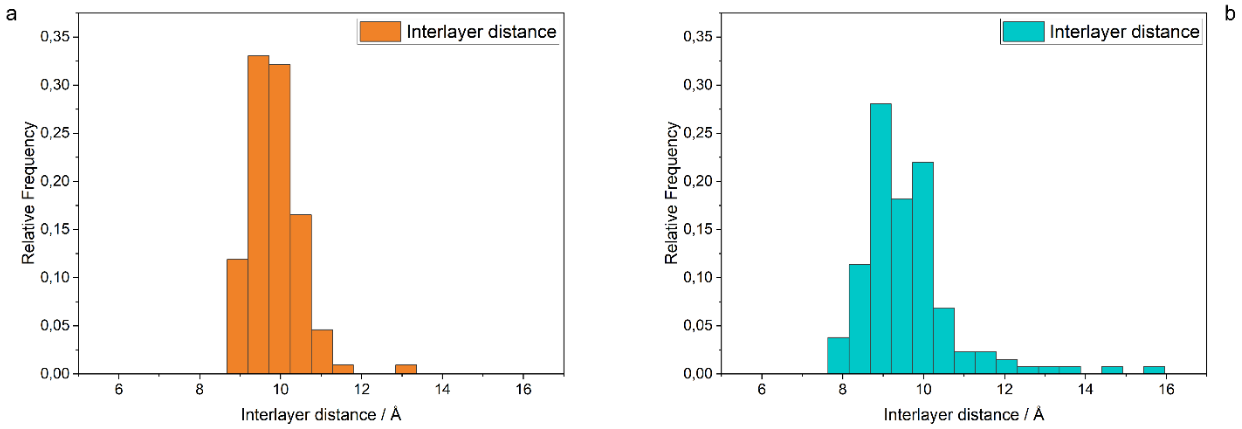

Figure S7 – distribution of the interlayer distances obtained from analysis of the TEM images reported in Figure 5 and Figure S6 for MXT-5 (a) and MXT-30 (b) compositions.

References

(1) Y. Mizuno, K. Sato, M. Mrinalini, T.S. Suzuki, Y. Sakka, Fabrication of textured Ti_3AlC_2 by spark plasma sintering and their anisotropic mechanical properties, *J. Ceram. Soc. Japan* **2013**, *121*, 366–369.

(2) B. Anasori, Y. Gogotsi, 2D Metal Carbides and Nitrides (MXenes), ©Springer Nature Switzerland, **2019**.

(3) Y. Zou, Z.M. Sun, H. Hashimoto, S. Tada, Synthesis of high-purity polycrystalline Ti_3AlC_2 through pulse discharge sintering Ti/Al/TiC powders, *J. Alloys Compd*. **2008**, *456*, 456–460.

(4) A. Gentile, C. Ferrara, S. Tosoni, M. Balordi, S. Marchionna, F. Cernuschi, M.H. Kim, H.W. Lee, R. Ruffo, Enhanced Functional Properties of $Ti_3C_2T_x$ MXenes as Negative Electrodes in Sodium-Ion Batteries by Chemical Tuning, *Small Methods* **2020**, *4* (9), 2000314.

(5) J. Rodríguez-Carvajal, Recent advances in magnetic structure determination by neutron powder diffraction, *Phys. B Phys. Condens. Matter*. **1993**, *192*, 55–69.

(6) M. Casas-Cabanas, J. Rikarte-Ormazabal, M. Reynaud, J. Rodríguez-Carvajal, FAULTS manual, 2015.

(7) M. Casas-Cabanas, M. Reynaud, J. Rikarte, P. Horbach, J. Rodríguez-Carvajal, FAULTS: A program for refinement of structures with extended defects, *J. Appl. Crystallogr.* **2016**, *49*, 2259–2269.

(8) B. Ravel, M. Newville, ATHENA, ARTEMIS, HEPHAESTUS: Data analysis for X-ray absorption spectroscopy using IFEFFIT, *J. Synchrotron Radiat.* **2005**, *12*, 537–541.

(9) M. Newville, IFEFFIT: Interactive XAFS analysis and FEFF fitting, *J. Synchrotron Rad*. **2001**, **8**, 322 – 324.

(10) B. Anasori, M.R. Lukatskaya, Y. Gogotsi, 2D metal carbides and nitrides (MXenes) for energy storage, *Nat. Rev. Mater*. **2017**, *2*, 16098.

(11) M. Naguib, M. Kurtoglu, V. Presser, J. Lu, J. Niu, M. Heon, L. Hultman, Y. Gogotsi, M.W. Barsoum, Two-dimensional nanocrystals produced by exfoliation of Ti_3AlC_2, *Adv. Mater*. **2011**, *23*, 4248–4253.

(12) Y. Bai, N. Srikanth, C.K. Chua, K. Zhou, Density Functional Theory Study of $M_{n+1}AX$ n Phases: A Review, *Crit. Rev. Solid State Mater. Sci*. **2019**,*44*, 56–107.

(13) J. Haemers, R. Gusmão, Z. Sofer, Synthesis Protocols of the Most Common Layered Carbide and Nitride MAX Phases, *Small Methods* **2020**, *4* (3), 1–32.

(14) C. Hu, H. Zhang, F. Li, Q. Huang, Y. Bao, New phases' discovery in MAX family, *Int. J. Refract. Met. Hard Mater*. **2013**, *36*, 300–312.

(15) S.R.G. Christopoulos, P.P. Filippatos, M.A. Hadi, N. Kelaidis, M.E. Fitzpatrick, A. Chroneos, Intrinsic defect processes and elastic properties of Ti_3AC_2 (A = Al, Si, Ga, Ge, In, Sn) MAX phases, *J. Appl. Phys*. **2018**, *123*, 025103.

(16) K. Akter, F. Parvin, M.A. Hadi, A.K.M.A. Islam, Insights into the predicted Hf_2SN in comparison with the synthesized MAX phase Hf_2SC: A comprehensive study, *Comput. Condens. Matter*. **2020**, *24*, e00485.

(17) M.A. Hadi, M.A. Rayhan, S.H. Naqib, A. Chroneos, A.K.M.A. Islam, Structural, elastic, thermal and lattice dynamic properties of new 321 MAX phases, *Comput. Mater. Sci.* **2019**, *170*, 109144.

(18) M. Benchakar, L. Loupias, C. Garnero, T. Bilyk, C. Morais, C. Canaff, N. Guignard, S. Morisset, H. Pazniak, S. Hurand, P. Chartier, J. Pacaud, V. Mauchamp, M.W. Barsoum, A. Habrioux, S. Célérier, One MAX phase, different MXenes: A guideline to understand the crucial role of etching conditions on $Ti_3C_2T_x$ surface chemistry, *Appl. Surf. Sci.* **2020**, *530*, 147209.

(19) M. Alhabeb, K. Maleski, B. Anasori, P. Lelyukh, L. Clark, S. Sin, Y. Gogotsi, Guidelines for Synthesis and Processing of Two-Dimensional Titanium Carbide ($Ti_3C_2T_x$ MXene), *Chem. Mater*. **2017**, *29* (18) 7633–7644.

(20) Y. Gogotsi, B. Anasori, The Rise of MXenes, *ACS Nano*. **2019**, *13* (8), 8491–8494.

(21) X. Li, M. Li, Q. Yang, G. Liang, Z. Huang, L. Ma, D. Wang, F. Mo, B. Dong, Q. Huang, C. Zhi, In Situ Electrochemical Synthesis of MXenes without Acid/Alkali Usage in/for an Aqueous Zinc Ion Battery, *Adv. Energy Mater*. **2020**, *10* (36), 202001791.

(22) A. Feng, Y. Yu, Y. Wang, F. Jiang, Y. Yu, L. Mi, L. Song, Two-dimensional MXene Ti_3C_2 produced by exfoliation of Ti_3AlC_2, *Mater. Des*. **2017**, *114*, 161–166.

(23) C. Ferrara, A. Gentile, S. Marchionna, R. Ruffo, $Ti_3C_2T_x$ MXene compounds for electrochemical energy storage, *Curr. Opin. Electrochem*. **2021**, *29*, 100764.

(24) Y. Cao, C. Guo, Y. Zou, Rapid synthesis of MXenes at room temperature, *Mater. Sci. Technol.* **2019**, *35*, 1904–1907.

(25) A. Pazniak, P. Bazhin, N. Shplis, E. Kolesnikov, I. Shchetinin, A. Komissarov, J. Polcak, A. Stolin, D. Kuznetsov, $Ti_3C_2T_x$ MXene characterization produced from SHS-ground Ti_3AlC_2, *Mater. Des.* **2019**, *183*, 108143.

(26) K. Goc, W. Prendota, L. Chlubny, T. Strączek, W. Tokarz, P.B. (Chachlowska), K.W. (Chabior), M.M. Bućko, J. Przewoźnik, J. Lis, Structure, morphology and electrical transport properties of the Ti_3AlC_2 materials, *Ceram. Int.* **2018**, *44*, 18322–18328.

(27) L.W. Finger, D.E. Cox, A.P. Jephcoat, Correction for powder diffraction peak asymmetry due to axial divergence, *J. Appl. Crystallogr*. **1994**, *27*, 892–900.

(28) J. Halim, K.M. Cook, M. Naguib, P. Eklund, Y. Gogotsi, J. Rosen, M.W. Barsoum, X-ray photoelectron spectroscopy of select multi-layered transition metal carbides (MXenes), *Appl. Surf. Sci.* **2016**, *362*, 406–417.

(29) C. Shi, M. Beidaghi, M. Naguib, O. Mashtalir, Y. Gogotsi, S.J.L. Billinge, Structure of nanocrystalline Ti_3C_2 MXene using atomic pair distribution function, *Phys. Rev. Lett.* **2013**, *112*, 125501.

(30) H. Wang, M. Naguib, K. Page, D.J. Wesolowski, Y. Gogotsi, Resolving the Structure of $Ti_3C_2T_x$ MXenes through Multilevel Structural Modeling of the Atomic Pair Distribution Function, *Chem. Mater.* **2016**, *28* (1), 349–359.

(31) R. Cheng, T. Hu, H. Zhang, C. Wang, M. Hu, J. Yang, C. Cui, T. Guang, C. Li, C. Shi, P. Hou, X. Wang, Understanding the Lithium Storage Mechanism of $Ti_3C_2T_x$ MXene, *J. Phys. Chem. C.* **2018**, *123*, 1099–1109.

(32) L.M. Dong, C. Ye, L.L. Zheng, Z.F. Gao, F. Xia, Two-dimensional metal carbides and nitrides (MXenes): preparation, property, and applications in cancer therapy, *Nanophotonics* **2020**, *9*, 2125–2145.

(33) J. Halim, I. Persson, E.J. Moon, P. Kühne, V. Darakchieva, P.O.Å. Persson, P. Eklund, J. Rosen, M.W. Barsoum, Electronic and optical characterization of 2D Ti_2C and Nb_2C (MXene) thin films, *J. Phys. Condens. Matter*. **2019**, *31* (16), 165301.

(34) L.H. Karlsson, J. Birch, J. Halim, M.W. Barsoum, P.O.Å. Persson, Atomically Resolved Structural and Chemical Investigation of Single MXene Sheets, *Nano Lett*. **2015**, *15* (8), 4955–4960.

(35) M. Magnuson, L.Å. Näslund, Local chemical bonding and structural properties in Ti_3AlC_2 MAX phase and $Ti_3C_2T_x$ MXene probed by Ti 1s X-ray absorption spectroscopy, *Phys. Rev. research* **2020**, *2,* 033516.

(36) S. Hu, S. Li, W. Xu, J. Zhang, Y. Zhou, Z. Cheng, Rapid preparation, thermal stability and electromagnetic interference shielding properties of two-dimensional Ti_3C_2 MXene, *Ceram. Int.* **2019**, *45*, 19902–19909.

(37) H. Zhang, G. Yang, X. Zuo, H. Tang, Q. Yang, G. Li, Computational studies on the structural, electronic and optical properties of graphene-like MXenes (M_2CT_2, M = Ti, Zr, Hf; T = O, F, OH) and their potential applications as visible-light driven photocatalysts, *J. Mater. Chem. A*. **2016**, *4*, 12913–12920.

(38) B. Anasori, C. Shi, E.J. Moon, Y. Xie, C.A. Voigt, P.R.C. Kent, S.J. May, S.J.L. Billinge, M.W. Barsoum, Y. Gogotsi, Control of electronic properties of 2D carbides (MXenes) by manipulating their transition metal layers, *Nanoscale Horizons.* **2016**, *1*, 227–234.

(39) B. Akgenc, New predicted two-dimensional MXenes and their structural, electronic and lattice dynamical properties, *Solid State Commun.* **2019**, *303–304*, 113739.

(40) M.R. Lukatskaya, S.M. Bak, X. Yu, X.Q. Yang, M.W. Barsoum, Y. Gogotsi, Probing the Mechanism of High Capacitance in 2D Titanium Carbide Using in Situ X-Ray Absorption Spectroscopy, *Adv. Energy Mater*. **2015**, *5* (15), 1500589.

(41) Y. Xie, M. Naguib, V.N. Mochalin, M.W. Barsoum, Y. Gogotsi, X. Yu, K.W. Nam, X.Q. Yang, A.I. Kolesnikov, P.R.C. Kent, Role of surface structure on li-ion energy storage capacity of two-dimensional transition-metal carbides, *J. Am. Chem. Soc*. **2014**, *136* (17), 6385–6394. doi:10.1021/ja501520b.

(42) M. Magnuson, M. Mattesini, Chemical bonding and electronic-structure in MAX phases as viewed by X-ray spectroscopy and density functional theory, *Thin Solid Films*. **2017**, *621*, 108–130.

(43) E.A. Stern, Number of relevant independent points in x-ray-absorption fine-structure spectra, *Phys. Rev. B*. **1993**, *48,* 9825–9827.

(43) T. Hu, M. Hu, B. Gao, W. Li, X. Wang, Screening Surface Structure of MXenes by High-Throughput Computation and Vibrational Spectroscopic Confirmation, *J. Phys. Chem. C*. **2018**, *122*, 18501–18509.

(44) Q. Tang, Z. Zhou, P. Shen, Are MXenes promising anode materials for Li ion batteries? Computational studies on electronic properties and Li storage capability of Ti_3C_2 and $Ti_3C_2X_2$ (X = F, OH) monolayer, *J. Am. Chem. Soc*. **2012**, *134* (40), 16909–16916.

(45) M. Naguib, V.N. Mochalin, M.W. Barsoum, Y. Gogotsi, 25th anniversary article: MXenes: A new family of two-dimensional materials, *Adv. Mater*. **2014**, *26* (7), 992–1005.

(47) C. Zhan, W. Sun, Y. Xie, D.E. Jiang, P.R.C. Kent, Computational Discovery and Design of MXenes for Energy Applications: Status, Successes, and Opportunities, *ACS Appl. Mater. Interfaces.* **2019**, *11* (28), 24885–24905.

(48) M. Khazaei, A. Ranjbar, M. Arai, T. Sasaki, S. Yunoki, Electronic properties and applications of MXenes: a theoretical review, *J. Mater. Chem. C.* **2017**, *5*, 2488–2503.

(49) J. Pang, R.G. Mendes, A. Bachmatiuk, L. Zhao, H.Q. Ta, T. Gemming, H. Liu, Z. Liu, M.H. Rummeli, Applications of 2D MXenes in energy conversion and storage systems, *Chem. Soc. Rev.* **2019**, *48*, 72–133.

(50) M. Khazaei, M. Arai, T. Sasaki, M. Estili, Y. Sakka, Two-dimensional molybdenum carbides: Potential thermoelectric materials of the MXene family, *Phys. Chem. Chem. Phys.* **2014**, *16*, 7841–7849.

(51) B. Lanson, Modelling of X-ray diffraction profiles: Investigation of defective lamellar structure crystal chemistry, *Layer. Miner. Struct. Their Appl. Adv. Technol*. **2012**, *11*, 151–202.

(52) B.E. Warren, Diffraction in solids, Dover Publications, **1990.**

(53) J. Serrano-Sevillano, M. Reynaud, A. Saracibar, T. Altantzis, S. Bals, G. Van Tendeloo, M. Casas-Cabanas, Enhanced electrochemical performance of Li-rich cathode materials through microstructural control, *Phys. Chem. Chem. Phys.* **2018**, *20*, 23112–23122.

(54) C. Delmas, C. Tessier, Stacking faults in the structure of nickel hydroxide: A rationale of its high electrochemical activity, *J. Mater. Chem.* **1997**, *7*, 1439–1443.

(55) M. Casas-Cabanas, J. Rodríguez-Carvajal, J. Canales-Vázquez, Y. Laligant, P. Lacorre, M.R. Palacín, Microstructural characterisation of battery materials using powder diffraction data: DIFFaX, FAULTS and SH-FullProf approaches, *J. Power Sources*. **2007**, *174* (2), 414–420.
